# Endothelial Notch1 signaling in white adipose tissue promotes cancer cachexia

**DOI:** 10.1038/s43018-023-00622-y

**Published:** 2023-09-25

**Authors:** Jacqueline Taylor, Leonie Uhl, Iris Moll, Sana Safatul Hasan, Lena Wiedmann, Jakob Morgenstern, Benedetto Daniele Giaimo, Tobias Friedrich, Elisenda Alsina-Sanchis, Francesca De Angelis Rigotti, Ronja Mülfarth, Sarah Kaltenbach, Darius Schenk, Felix Nickel, Thomas Fleming, David Sprinzak, Carolin Mogler, Thomas Korff, Adrian T. Billeter, Beat P. Müller-Stich, Mauricio Berriel Diaz, Tilman Borggrefe, Stephan Herzig, Maria Rohm, Juan Rodriguez-Vita, Andreas Fischer

**Affiliations:** 1 Division Vascular Signaling and Cancer, German Cancer Research Center (DKFZ), Heidelberg, Germany; 2 Theodor Boveri Institute, Department of Biochemistry and Molecular Biology, Biocenter, University of Würzburg, Würzburg, Germany; 3 Department of Clinical Chemistry, University Medical Center Göttingen, Göttingen, Germany; 4 Department of Internal Medicine Endocrinology and Clinical Chemistry, University of Heidelberg, Heidelberg, Germany; 5 Institute of Biochemistry, University of Giessen, Giessen, Germany; 6 Biomedical Informatics and Systems Medicine, Science Unit for Basic and Clinical Medicine, Giessen, Germany; 7 Tumor-Stroma Communication Laboratory, Centro de Investigación Príncipe Felipe, Valencia, Spain; 8 Department of General, Visceral and Transplantation Surgery, University of Heidelberg, Heidelberg, Germany; 9 German Center of Diabetes Research (DZD), Neuherberg, Germany; 10 School of Neurobiology, Biochemistry and Biophysics, George S. Wise Faculty of Life Sciences, Tel Aviv University, Tel Aviv, Israel; 11 Institute of Pathology, Technical University of Munich School of Medicine, Technical University of Munich, Munich, Germany; 12 Institute of Physiology and Pathophysiology, Department of Cardiovascular Physiology, University of Heidelberg, Heidelberg, Germany; 13 European Center for Angioscience (ECAS), Medical Faculty Mannheim, University of Heidelberg, Mannheim, Germany; 14 Institute for Diabetes and Cancer, Helmholtz Center Munich, German Center for Diabetes Research (DZD), Neuherberg, Germany; 15 Joint Heidelberg–IDC Translational Diabetes Unit, Department of Inner Medicine I, Heidelberg University Hospital, Heidelberg, Germany; 16 Chair Molecular Metabolic Control, Technical University of Munich, Munich, Germany; 17 German Center for Cardiovascular Research (DZHK), partner site Göttingen, Göttingen, Germany

**Keywords:** Cancer, Cancer, Cancer therapy, Cancer metabolism

## Abstract

Cachexia is a major cause of morbidity and mortality in individuals with cancer and is characterized by weight loss due to adipose and muscle tissue wasting. Hallmarks of white adipose tissue (WAT) remodeling, which often precedes weight loss, are impaired lipid storage, inflammation and eventually fibrosis. Tissue wasting occurs in response to tumor-secreted factors. Considering that the continuous endothelium in WAT is the first line of contact with circulating factors, we postulated whether the endothelium itself may orchestrate tissue remodeling. Here, we show using human and mouse cancer models that during precachexia, tumors overactivate Notch1 signaling in distant WAT endothelium. Sustained endothelial Notch1 signaling induces a WAT wasting phenotype in male mice through excessive retinoic acid production. Pharmacological blockade of retinoic acid signaling was sufficient to inhibit WAT wasting in a mouse cancer cachexia model. This demonstrates that cancer manipulates the endothelium at distant sites to mediate WAT wasting by altering angiocrine signals.

## Abstract (web summary)

Taylor et al. show that tumor cells promote white adipose tissue (WAT) wasting and cachexia by overactivation of Notch1 signaling and retinoic acid production in distant WAT endothelium, which can be therapeutically targeted to inhibit wasting.

## Main

Cancer cachexia is a multifactorial wasting syndrome that affects the majority of individuals with advanced cancer. Loss of muscle and fat mass is accompanied by decreased quality of life, poor response to chemotherapy and high mortality. Still, no standardized treatment exists^1^.

Cachexia development progresses in three stages: precachexia, cachexia and refractory cachexia. Although cachexia has been defined as >5% weight loss within a 6-month time span, systemic metabolic alterations, including perturbed glucose and lipid metabolism, can already be detected in individuals during the precachectic state^2^. In particular, white adipose tissue (WAT) remodeling already occurs during precachexia, leading to impaired lipid metabolism, macrophage infiltration, chronic inflammation and eventually fibrosis^3–6^.

WAT wasting is driven by combinatorial action of tumor-secreted and/or host-secreted factors, such as tumor necrosis factor-α (TNF-α), interleukin-6 (IL-6) or IL-1β, which are transported through the bloodstream^6,7^. We hypothesized that these circulating factors act first on the continuous endothelium at distant organs, such as WAT, and that the endothelium would mediate their effects.

Endothelial cells (ECs) form the inner lining of blood vessels. The continuous endothelial layer prevents free diffusion of procachectic proteins into the tissue in most organs. ECs provide a large surface area and may act as first responders at the blood–tissue interface, relaying responses and instructive cues to neighboring cells^8^. Such angiocrine functions operate in an organ-specific manner and are essential for development and control of organ metabolism and tumor progression. Within a solid tumor mass, ECs play essential roles. ECs are building blocks for new blood vessels that nourish the tumor and also control tumor progression and metastasis through angiocrine factors^9,10^. In particular, the large surface area of the endothelium enables it to amplify tumor-derived signals at distant sites, as shown for metastatic spreading^11,12^. Based on this, we addressed the question of whether tumors located at distant sites induce WAT remodeling by changing the angiocrine landscape in adipose tissue ECs (AT-ECs), thereby orchestrating critical aspects of cancer cachexia.

## Results

### AT-EC Notch1 signaling is overactive in precachexia

**Fig. 1:**
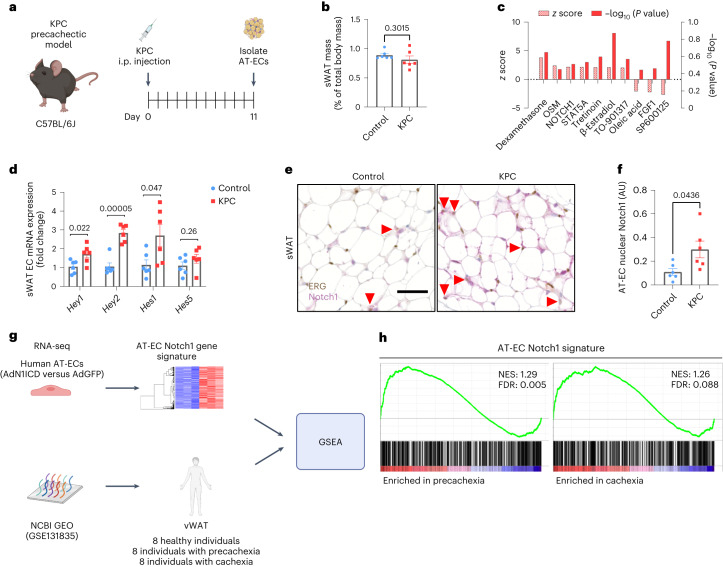
Tumors induce Notch1 overactivation in the adipose tissue endothelium. **a**, Timeline of AT-EC isolation from precachectic mice injected intraperitoneally (i.p.) with KPC pancreatic adenocarcinoma cells. **b**, Relative mass of sWAT collected from KPC precachectic mice (*n* = 6 animals per group). **c**, Top ten IPA-predicted upstream regulators of transcriptomic changes in sWAT AT-ECs from precachectic versus non-tumor-bearing mice. Plotted are *z* scores and –log_10_ (*P* values); *n* = 3–4. **d**, mRNA levels of prototypical Notch target genes and signaling components in precachectic AT-ECs (*n* = 6 animals per group). **e**, Representative images of ERG (DAB, brown) and Notch1 (AP, red) co-stainings comparing sWAT from PBS-injected (control) and KPC-injected mice; scale bar, 50 µm. **f**, Quantification of ERG^+^Notch1^+^ ECs in sWAT (*n* = 6 animals per group, ten images averaged per mouse); AU, arbitrary units. **g**, Enrichment of an ‘AT-EC Notch1 gene signature’ was analyzed in publicly available datasets from whole vWAT biopsies from healthy individuals and individuals with precachexia and cachexia (GSE131835). **h**, Enrichment plots of the ‘AT-EC Notch1 gene signature’ comparing precachectic and cachectic vWAT to healthy vWAT; NES, normalized enrichment score; FDR, false discovery rate. Data shown represent mean ± s.e.m. Data were analyzed by unpaired, two-sided *t*-test with Welch correction. Experiments in **b** and **d** were performed twice with consistent results. Results shown are from one representative experiment. Source data

**Extended Data Fig. 1:**
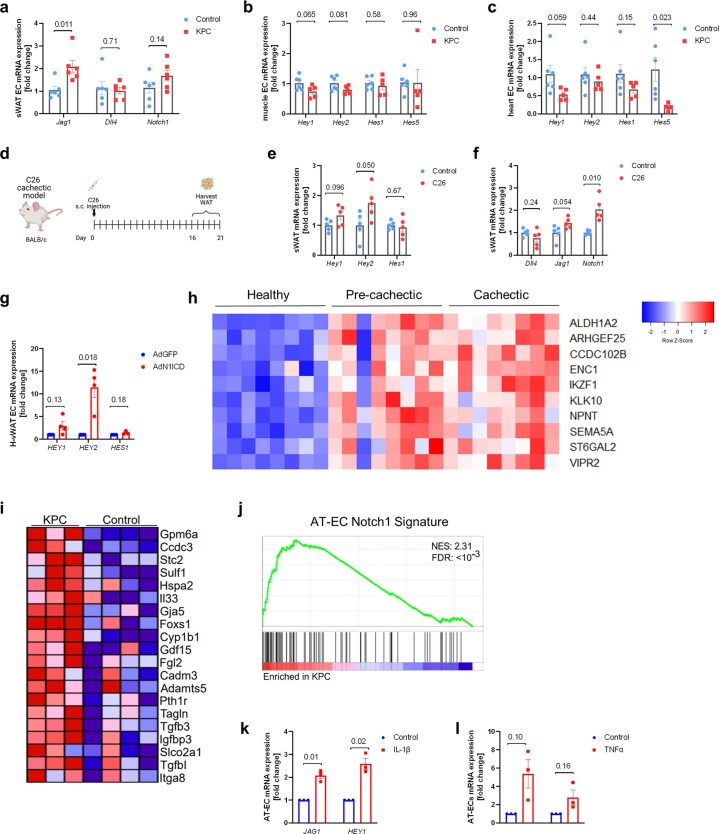
Notch signalling is overactive in human and murine models of cancer cachexia. **a**, RT-qPCR analysis of Notch1 ligands *Jag1* and *Dll4* as well as total *Notch1* in sWAT ECs isolated from pre-cachectic KPC mice (n = 6 animals per group). **b**,**c**, RT-qPCR analysis of prototypical Notch1 target genes in muscle ECs (**b**) and heart ECs (**c**) of KPC mice compared to non-tumour bearing controls (n = 5-6 animals per group). **d**, Analysis scheme of whole tissue harvested from C26 cachectic mice. **e**, RT-qPCR analysis of prototypical Notch1 target genes and **f**, Notch ligands, *Jag1, Dll4*, and total *Notch1* in whole sWAT harvested from C26 mice (n = 6 animals per group). **g**, RT-qPCR of prototypical Notch1 target genes in human vWAT ECs overexpressing AdN1ICD or AdGFP (n = 3 (*HES1*) or 4 (*HEY1*, *HEY2*) biologically independent experiments). **h**, Heatmap comparing expression levels of the top 10 leading edge genes of the ‘AT-EC Notch1 gene signature’ in patient samples (GSE131835). **i**, Heatmap of the top 20 leading edge genes of the ‘AT-EC Notch1 gene signature’ in KPC sWAT ECs isolated from mice during pre-cachexia and the corresponding **j**, GSEA enrichment plot. **k**, Human AT-ECs treated with recombinant IL-1β for 24 hours were analysed for *JAG1* and *HEY1* mRNA levels (n = 3 biologically independent experiments). **l**, Human AT-ECs treated with recombinant TNFα for 6 hours were analysed for *JAG1*and *HEY1* mRNA levels (n = 3 biologically independent experiments). Data shown represent mean ± SEM, unpaired two-sided t-test with Welch correction. Experiments shown in **a, b** and **c** were performed twice with consistent results. Results shown are from one representative experiment. Source data

To investigate transcriptomic changes in the WAT endothelium during precachexia, we used the KPC pancreatic adenocarcinoma (PDAC) cachexia mouse model (Fig. 1a). Subcutaneous WAT (sWAT) fat pads were excised at a time point when no differences in body weight and WAT mass compared to age-matched non-tumor-bearing mice were yet observed (Fig. 1b). RNA profiles from isolated AT-ECs at this precachectic state were compared by microarray analysis. Ingenuity Pathway Analysis (IPA) predicted Notch1 as a top upstream regulator of transcriptomic changes in KPC AT-ECs (Fig. 1c). Notch1, a master regulator of angiogenesis and angiocrine signaling^13^, is frequently overactivated in tumor ECs and in ECs within the premetastatic niche^11,14^. Quantitative PCR with reverse transcription (RT–qPCR) confirmed upregulation of prototypical Notch1 target genes (Fig. 1d) and the gene encoding the Notch ligand JAG1 in AT-ECs (Extended Data. Fig. 1a) but not in muscle ECs (Extended Data Fig. 1b,c). Elevated *Hey2*, *Jag1* and *Notch1* expression was also observed in whole sWAT fat pads during cachexia in C26 colorectal tumor-bearing mice (Extended Data Fig. 1d–f), showing that this genetic program is not restricted to PDAC. Coimmunostainings of the nuclear EC marker ERG and nuclear Notch1 in sWAT from KPC mice further validated increased active Notch1 signaling in AT-ECs (Fig. 1e,f).

Next, we assessed whether cancer cachexia in humans is also linked to increased endothelial Notch1 signaling (Fig. 1g). We therefore established a Notch1-induced gene signature by identifying the top 500 upregulated genes in AT-ECs isolated from human visceral WAT (vWAT), which expressed constitutively active Notch1-intracellular domain (AdN1ICD) or green fluorescent protein (GFP; AdGFP) as a control (Extended Data Fig. 1g). Gene set enrichment analysis (GSEA) showed that the ‘AT-EC Notch1 gene signature’ was highly enriched in a gene expression dataset (Gene Expression Omnibus (GEO) GSE131835) obtained from vWAT samples from individuals with cachexia with oesophago-gastric cancer and from individuals with precachexia and stable weight compared to vWAT samples from healthy, cancer-free donors (Fig. 1h and Extended Data Fig. 1h)^15^, indicating a potential role for EC Notch1 signaling in human cachectic phenotypes. An ‘AT-EC Notch1 gene signature’ prepared from human sWAT ECs was also confirmed to be significantly enriched in KPC sWAT ECs (Extended Data Fig. 1i,j), thus further validating increased expression of Notch1 signaling targets during precachexia.

As proinflammatory cytokines have been shown to upregulate Notch ligands, in particular JAG1, in ECs^16^, we treated human AT-ECs with well-established cachexokines. Similar to studies in human umbilical vein ECs^17^, IL-1β and TNF-α upregulated the expression of the Notch ligand *JAG1* and Notch target gene *HEY1* (Extended Data Fig. 1k,l). In addition, when comparing blood sera from KPC tumor-bearing mice to sera from tumor-free control mice, we observed that circulating TNF-α protein levels increased in tumor-bearing mice (Supplementary Table 1). This suggests that proinflammatory cytokines, such as TNF-α, produced in response to tumor growth and circulating through the bloodstream may enforce endothelial Notch1 signaling at distant sites.

### Sustained AT-EC Notch1 signaling drives WAT remodeling

**Extended Data Fig. 2:**
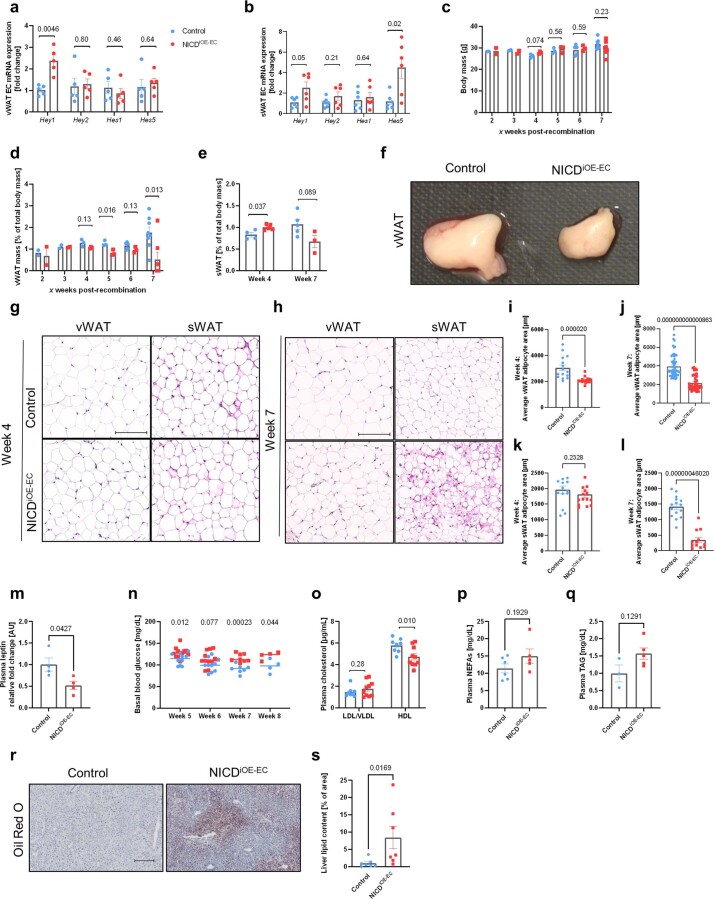
Chronic overactivation of EC Notch1 signalling induces lipodystrophy in male NICD^iOE-EC^ mice. **a**,**b**, RT-qPCR analysis of Notch1 target genes and ligands in male NICD^iOE-EC^ AT-ECs isolated from vWAT (n = 6 animals per group; **a**) and sWAT (**b**) two weeks after tamoxifen treatment (n = 6 animals per group). **c**,**d**, Body (**c**) and relative vWAT mass (**d**) of NICD^iOE-EC^ male mice 2-7 weeks post-recombination (n = 2-8 animals per group). **e**, Relative sWAT mass at weeks 4 and 7 in NICD^iOE-EC^ male mice (n = 3-5 animals per group). **f**, Representative photograph of NICD^iOE-EC^ vWAT at week 6 post-recombination. **g**,**h**, Representative images of vWAT and sWAT H&E stainings at weeks 4 (**g**) and 7 (**h**) from one (week 4) or two (week 7) independent experiments. Scale bar: 200 µm. **i**–**l**, Quantification of vWAT (n = 3-12 animals per group, 5 images per mouse; **i** and **j**) and sWAT (**k** and **l**) adipocyte area at weeks 4 (**i** and **k**) and 7 (**j** and **l**) quantified using the Adiposoft plugin for ImageJ (n = 3 or 5 animals per group, 5 images per mouse). **m**, Relative plasma leptin levels measured at week 7 by adipokine array (n = 4 animals per group). **n**, Basal blood glucose levels of male NICD^iOE-EC^ mice between weeks 5 and 8 (n = 4-11 animals per group). **o**, Plasma LDL/VLDL and HDL cholesterol (n = 8-10 animals per group). **p**,**q**, NEFA (n = 5-6 animals per group; **p**) and TAG (n = 3-5 animals per group; **q**) measured in male NICD^iOE-EC^ mice at week 7. **r**, Livers were stained with Oil Red O at week 7 when substantial fat loss was observed. Scale bar: 200 µM**. s**, Lipid droplet content was quantified from Oil Red O staining as percentage of total area (n = 7 animals per group). Data shown represent mean ± SEM, unpaired two-sided t-test with Welch correction (a-e, k, m-q) or Mann-Whitney test (i-l, s). Data in **i** and **j** were pooled from two individual experiments. Source data

Next, we examined whether overactivation of AT-EC Notch1 signaling could alone (that is, without the presence of a tumor) induce adipose tissue remodeling as usually seen in cancer cachexia. We used a very well-characterized Notch1 gain-of-function mouse model (NICD^iOE-EC^) in which constitutively active N1ICD is expressed under the highly EC-specific tamoxifen-inducible *Cdh5* (vascular endothelial cadherin) promoter^11,18,19^. Recombination was induced in adult mice. AT-ECs isolated from male NICD^iOE-EC^ mice showed moderate overexpression of classical Notch1 targets (Extended Data Fig. 2a,b), which was comparable to AT-ECs from precachectic mice (Fig. 1d) and similar to levels observed in other vascular beds^11,18^. Although no changes in body mass were observed (Extended Data Fig. 2c), male NICD^iOE-EC^ mice showed a gradual loss of WAT mass (Extended Data Fig. 2d–f) and decreased adipocyte size (Extended Data Fig. 2g–l).

Cachexia is often accompanied by insulin resistance^1,20,21^, which we previously observed in NICD^iOE-EC^ mice^18^. Metabolic profiling revealed lower leptin levels, increased basal blood glucose, altered lipoprotein cholesterol levels and slightly augmented plasma triacylglycerol (TAG) and non-esterified fatty acid (NEFA) levels, reflecting inadequate lipid storage (Extended Data Fig. 2m–q). Consistently, ectopic fat deposition was observed in livers following WAT loss (Extended Data Fig. 2r,s).

### Notch1 signaling drives remodeling in a sex-specific manner

**Extended Data Fig. 3:**
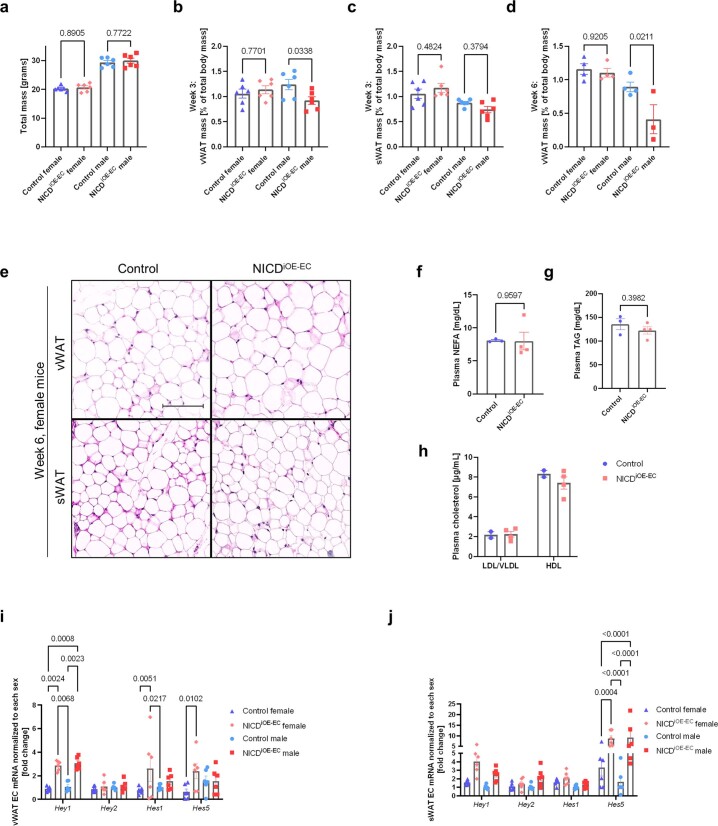
Male but not female NICD^iOE-EC^ mice are lipodystrophic at the timepoints analysed. **a**–**c**, Total body (**a**), vWAT (**b**) and sWAT (**c**) mass were compared in male and female NICD^iOE-EC^ mice at 3 weeks post-recombination (n = 6 animals per group). **d**, vWAT mass was also compared at 6 weeks (n = 3-4 animals per group). **e**, Representative images of vWAT and sWAT H&E staining from female NICD^iOE-EC^ mice at week 6. Scale bar: 100 µM. **f**, Plasma NEFA (n = 3-4 animals per group). **g**,**h**, TAG (n = 3-4 animals per group; **g**) and LDL/VLDL and HDL cholesterol levels (n = 2-4 animals per group; **h**) in female NICD^iOE-EC^ mice. **i**,**j**, RT-qPCR comparison of prototypical Notch1 target genes in male and female vWAT (**i**) and sWAT (**j**) AT-ECs at 3 weeks post-recombination (n = 6 animals per group). Data shown represent mean ± SEM, 1-way ANOVA with Tukey’s test (**a**–**d**), unpaired two-sided t-test with Welch correction (**f**–**h**) or 2-way ANOVA (**i** and **j**). Experiments **i** and **j** were performed twice with consistent results. Shown is one representative experiment. Source data

Cachexia is often more severe in males than in females, both in humans and in animal models^22–24^. When comparing the WAT phenotype of male versus female NICD^iOE-EC^ mice, we observed that, contrary to males, WAT mass and adipocyte morphology remained unaltered in female NICD^iOE-EC^ mice (Extended Data Fig. 3a–e). No changes were observed in NEFA, TAG and lipoprotein cholesterol levels (Extended Data Fig. 3f–h). Notably, the expression of prototypical Notch1 target genes was similar in both male and female mice (Extended Data Fig. 3i,j), ruling out the possibility that such differences occurred solely due to different gene recombination efficiency. In summary, there are substantial sex-specific differences in tissue wasting in individuals with cancer and in mouse cancer cachexia models^22–24^, and such sex-specific discrepancies in phenotype could also be observed between male and female NICD^iOE-EC^ mice. Based on this observation, we used male mice for subsequent investigations to unravel the responsible mechanism.

### Beiging, apoptosis and fibrosis drive NICD^iOE-EC^ WAT loss

**Fig. 2:**
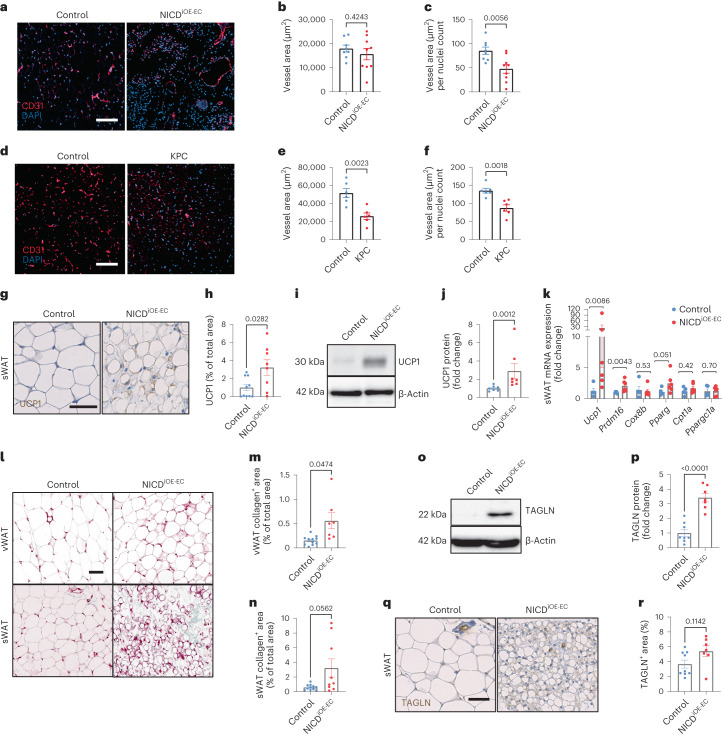
Beiging and fibrosis drive NICD^iOE-EC^ adipose tissue remodeling. **a**, Representative images of CD31 and DAPI staining of control and NICD^iOE-EC^ sWAT from two individual experiments; scale bar, 200 µm. **b**,**c**, Stainings were quantified based on vessel area (μm^2^; **b**) and vessel area (μm^2^) per nuclei count (**c**); *n* = 8–9 animals per group. **d**, Representative images of CD31 and DAPI staining of control and KPC sWAT from one individual experiment; scale bar, 200 µm. **e**,**f**, Stainings were quantified based on vessel area (μm^2^; **e**) and vessel area (μm^2^) per nuclei count (**f**); *n* = 6 biologically independent animals. **g**,**h**, Representative images from two individual experiments of UCP1 (DAB) staining of control and NICD^iOE-EC^ sWAT (**g**) and quantification (**h**) as a percentage of total area (*n* = 8–10 animals per group); scale bar, 50 µm. **i**, Representative western blot of UCP1 expression from two individual experiments from whole control and NICD^iOE-EC^ sWAT. **j**, UCP1 western blot quantification was normalized to β-actin (*n* = 7 animals per group). **k**, mRNA levels of thermogenic and/or beiging markers in whole sWAT (*n* = 5–6 animals per group). **l**, Representative images from two individual experiments of Masson’s trichrome-stained vWAT and sWAT; scale bar, 100 µm. **m**,**n**, Quantification of collagen areas as a percentage of total section area excluding the reticular interstitium in vWAT (*n* = 7–11 animals per group; **m**) and sWAT (*n* = 9–11 animals per group; **n**). **o**, Representative western blot of TAGLN expression in lysates from whole control and NICD^iOE-EC^ sWAT. **p**, Quantifications were normalized to β-actin (*n* = 7–8 animals per group; *P* = 0.00004106). **q**,**r**, Representative images (**q**) from two individual experiments of TAGLN-stained (DAB) sWAT and quantification (**r**) of TAGLN^+^ area analyzed from whole sWAT sections; *n* = 7–9 animals per group; scale bar, 50 µm. Data shown represent mean ± s.e.m. and were analyzed by unpaired, two-sided *t*-test with Welch correction (**b**, **c**, **e**, **f**, **h** and **m**) or Mann–Whitney test (**j**, **k**, **n**, **p** and **r**). Experiments in **a**–**c, g**–**j** and **l**–**r** were performed twice, and results were pooled from two independent experiments. Results were consistent between the two experiments. Source data

**Extended Data Fig. 4:**
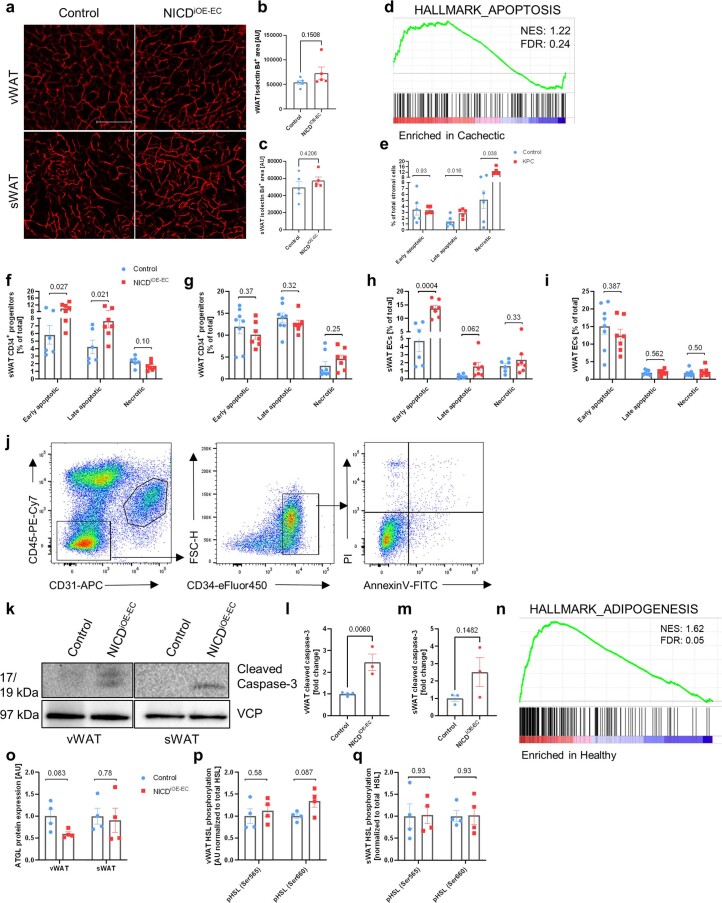
Apoptosis contributes to NICD^iOE-EC^ WAT loss in male mice. **a**, Representative images of isolectin B4 whole mount stainings of NICD^iOE-EC^ vWAT and sWAT at week 7 post-recombination. **b**,**c**, Isolectin B4^+^ endothelial coverage area was quantified (n = 5 animals per group). **d**, Enrichment plot of ‘Hallmark_apoptosis’ in cachectic compared to healthy vWAT (GSE131835). **e**, Adipose stromal cells (CD45^-^CD31^-^CD140^+^Sca1^+^) assessed in sWAT of KPC mice compared to non-tumour bearing controls for early apoptotic (Annexin V^+^PI^−^), late apoptotic (Annexin V^+^PI^+^) and necrotic (Annexin V^−^PI^+^) markers by flow cytometry (n = 5-6 animals per group). **f**–**i**, Quantified percentages of apoptotic progenitors (CD45^-^CD31^−^CD34^+^; **f** and **g**) and AT-ECs (CD45^−^CD31^+^; **h** and **i**) in vWAT (**g** and **i**) and sWAT (**f** and **h**) of NICD^iOE-EC^ mice at week 7 analysed by flow cytometry (n = 6-8 animals per group). **j**, Gating strategy of CD45^−^CD31^−^CD34^+^ adipose progenitors to assess early apoptosis (Annexin V^+^PI^−^), late apoptosis (Annexin V^+^PI^+^) and necrosis (Annexin V^−^PI^+^) by flow cytometry. **k**, Western blots of cleaved caspase-3 from control and NICD^iOE-EC^ vWAT and sWAT. **l**,**m**, Quantifications of vWAT (**l**) and sWAT (**m**) cleaved caspase-3 levels normalized to VCP (n = 3-4 animals per group). **n**, Enrichment plot of ‘Hallmark_adipogenesis’ in healthy vWAT compared to cachectic patient samples (GSE131835). **o**, Western blot analyses of whole vWAT and sWAT ATGL protein levels normalized to the housekeeping protein VCP (n = 4 animals per group). **p**,**q**, Ser565 and Ser660 HSL phosphorylation and total HSL were quantified from whole sWAT and vWAT by Western blot analysis and normalized to the housekeeping protein VCP. The ratios of pHSL:HSL in vWAT (**p**) and sWAT (**q**) of NICD^iOE-EC^ mice at week 5 post-recombination were quantified (n = 4 animals per group). Data shown represent mean ± SEM, Mann –Whitney test (b, c) or unpaired two-sided t-test with Welch correction (e-q). Source data

Notch1 regulates angiogenesis and angiocrine signaling during development^13^ and prevents vascular malformations during adulthood^25^. As changes in WAT vascularization alter adipocyte metabolism^9^, we examined microvessel density and morphology of WAT from NICD^iOE-EC^ mice. We found no differences in vessel density reaching statistical significance (Fig. 2a,b and Extended Data Fig. 4a–c) when looking at vessel area alone. However, when analyzing vessel area normalized to the number of DAPI^+^ nuclei, vessel area was reduced in WAT (Fig. 2c). Such a reduction in vessel area could also be observed in KPC tumor-bearing mice compared to non-tumor controls (Fig. 2d–f), showing again that male NICD^iOE-EC^ mice phenocopy many features of WAT wasting as seen in classical cancer cachexia models.

It is known that the apoptosis rate in cells of WAT increases throughout cachexia progression^5^. In line with this, we observed enrichment of the ‘hallmark apoptosis’ gene set in human cachectic WAT samples (GSE131835; Extended Data Fig. 4d) and increased apoptotic and necrotic stromal cells in KPC WAT (Extended Data Fig. 4e). In NICD^iOE-EC^ mice, increased apoptosis of sWAT adipose progenitors (CD45^–^CD31^–^CD34^+^) and AT-ECs (Extended Data Fig. 4f–j) and increased levels of cleaved caspase-3 in both WAT depots (Extended Data Fig. 4k–m) contributed to loss of fat. We also observed reduced enrichment of the ‘hallmark adipogenesis’ gene set in cachectic WAT in humans (GSE131835; Extended Data Fig. 4n), which may, in part, be explained by increased apoptosis, as described in previous reports^5^.

To better understand the role of endothelial Notch1 in WAT wasting, we examined whether other key mediators of cachexia are present in NICD^iOE-EC^ WAT, including increased lipolysis, beiging and fibrosis. Protein levels of lipolytic proteins ATGL and phospho-HSL (Ser 565 and Ser 660) were unaltered (Extended Data Fig. 4o–q). However, expression of the gene encoding thermogenic regulator uncoupling protein 1 (UCP1) as well as protein levels were upregulated in whole sWAT (Fig. 2g–k) along with *Prdm16*, suggesting enhanced thermogenesis in NICD^iOE-EC^ WAT. This is similar to cancer models, which often show WAT beiging over the course of cachexia^26^.

**Extended Data Fig. 5:**
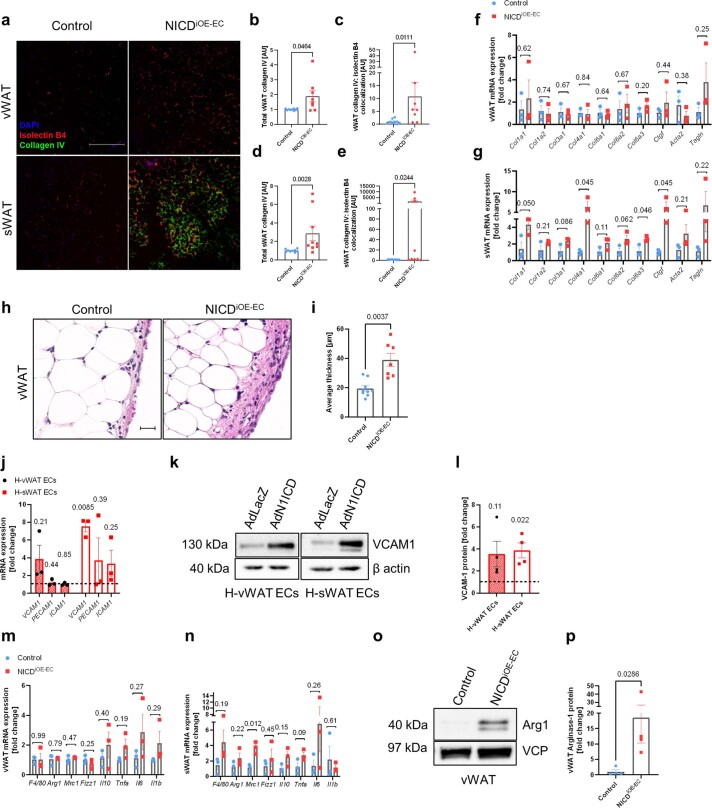
Enhanced collagen secretion and type 2 inflammation in NICD^iOE-EC^ WAT. **a**, Representative collagen IV, isolectin B4 and DAPI fluorescence stainings of NICD^iOE-EC^ vWAT and sWAT sections at week 7 post-recombination. Scale bar: 100 µM. **b**–**e**, vWAT (n = 8 biologically independent animals pooled from two independent experiments) and sWAT (n = 9 animals per group pooled from two independent experiments) were quantified for total collagen IV expression as well as collagen IV:isolectin B4 co-localization. **f**,**g**, RT-qPCR analysis of extracellular matrix and fibrotic markers in vWAT (**f**) and sWAT (**g**); n = 3 animals per group. **h**, Representative images of H&E stained vWAT reticular interstitium. Scale bar: 20 µm. **i**, Average thickness of the reticular interstitium from vWAT H&E staining (n = 7-8 animals per group pooled from two independent experiments). **j**, RT-qPCR analysis of adhesion molecules in human AT-ECs from vWAT and sWAT overexpressing AdN1ICD compared to AdLacZ-overexpressing controls (n = 3 biologically independent experiments). **k**,**l**, Western blots (**k**) of VCAM-1 levels in human AT-ECs were quantified (**l**) and normalized to β-actin (n = 4 biologically independent experiments). **m**,**n**, RT-qPCR analysis of macrophage markers and cytokines in whole vWAT (**m**) and sWAT (**n**) of NICD^iOE-EC^ mice at week 4 post-recombination (n = 3 animals per group). **o**,**p**, Western blot (**o**) of Arg-1 in whole NICD^iOE-EC^ vWAT at week 6 was quantified (**p**) relative to VCP (n = 4 animals per group). Data shown represent mean ± SEM, Mann-Whitney test (**b**–**e** and **p**) or unpaired two-sided t-test with Welch correction (**f**–**n**). Experiments **a**–**e** and **h** and **i** were performed twice and results were pooled. Results were consistent between the two experiments. Source data

WAT from NICD^iOE-EC^ mice displayed collagen accumulation (Fig. 2l–n and Extended Data Fig. 5a–e), increased expression of extracellular matrix components (Extended Data Fig. 5f,g) and severe thickening of the reticular interstitium (Extended Data Fig. 5h,i), an encapsulating layer rich in collagen and elastin^27^. Expression of the fibroblast marker transgelin (TAGLN) was also upregulated (Fig. 2o–r). Taken together, fibrosis is a contributor to WAT wasting in NICD^iOE-EC^ mice.

Fibrosis and excessive tissue repair can result from unresolved inflammation^28,29^. During cachexia, macrophages infiltrate WAT and contribute to a chronic inflammatory, hypermetabolic state^30^. Sustained endothelial Notch1 activity in other organs promotes myeloid cell infiltration through transcriptional induction of vascular cell adhesion molecule 1 (*VCAM1*)^11,31^. Increased VCAM1 expression was also confirmed in human AT-ECs (Extended Data Fig. 5j–l). Analysis of whole WAT showed increased gene expression of type 2 inflammatory macrophage markers mannose receptor (*Mrc1*) and arginase-1 (*Arg1*; Extended Data Fig. 5m–p), indicating a typical type 2 immune response in WAT^29^.

In summary, sustained AT-EC Notch1 activation promotes WAT beiging, apoptosis, fibrosis and type 2 inflammation, mimicking the hallmarks of the cachectic phenotype.

### Retinoic acid (RA) production is enhanced by EC Notch1 signaling

**Fig. 3:**
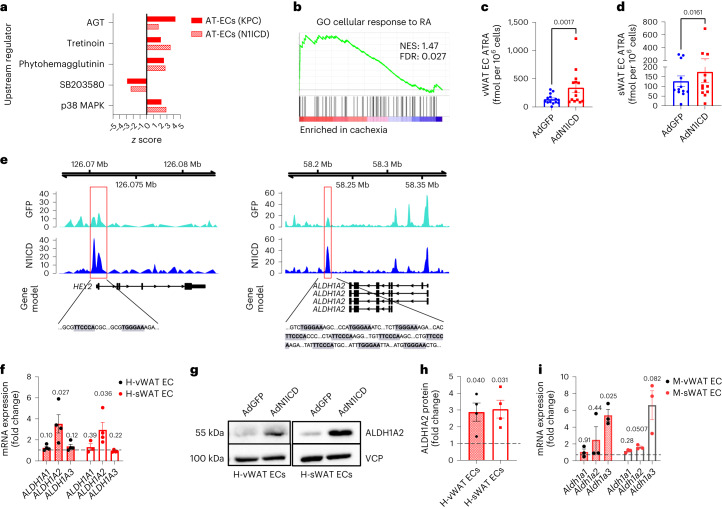
Notch1 regulates RA metabolism through ALDH1 expression. **a**, Overlapping predicted upstream regulators of transcriptomic changes in KPC (precachectic) and N1ICD-overexpressing AT-EC datasets compared to their respective controls. **b**, GO term ‘cellular response to RA’ enrichment plot of cachectic versus healthy vWAT (GSE131835). **c**,**d**, Intracellular ATRA levels in human vWAT ECs (*n* = 14 biologically independent experiments; **c**) and sWAT ECs (*n* = 12 biologically independent experiments; **d**) treated with AdN1ICD or AdGFP were measured by mass spectrometry. **e**, vWAT ECs overexpressing AdN1ICD or AdGFP were analyzed by ChIP–seq using an antibody to H3K27ac. N1CD increased H3K27ac at the *HEY2* locus (left, red box) and at the *ALDH1A2* locus (right, red box). RBP-J binding motifs are identified within the regions associated with increased H3K27ac after N1ICD overexpression at both the *HEY2* and *ALDH1A2* loci. RBP-J binding motifs are highlighted by the gray boxes; Mb, megabases. **f**,**g**, mRNA levels of *ALDH1* isozymes (*n* = 3–4 biologically independent experiments; **f**) and ALDH1A2 protein levels (**g**) analyzed by western blotting in human AT-ECs overexpressing AdN1ICD or AdGFP; H-vWAT, human vWAT; H-sWAT, human sWAT. **h**, Western blots were quantified and normalized to VCP (*n* = 4 biologically independent experiments). **i**, mRNA expression of *Aldh1* isozymes in NICD^iOE-EC^ AT-ECs (*n* = 3 biologically independent experiments). Data shown represent mean ± s.e.m. and were analyzed by Wilcoxon test (**c** and **d**) or unpaired, two-sided *t*-test with Welch correction (**f**, **h** and **i**). Source data

**Extended Data Fig. 6:**
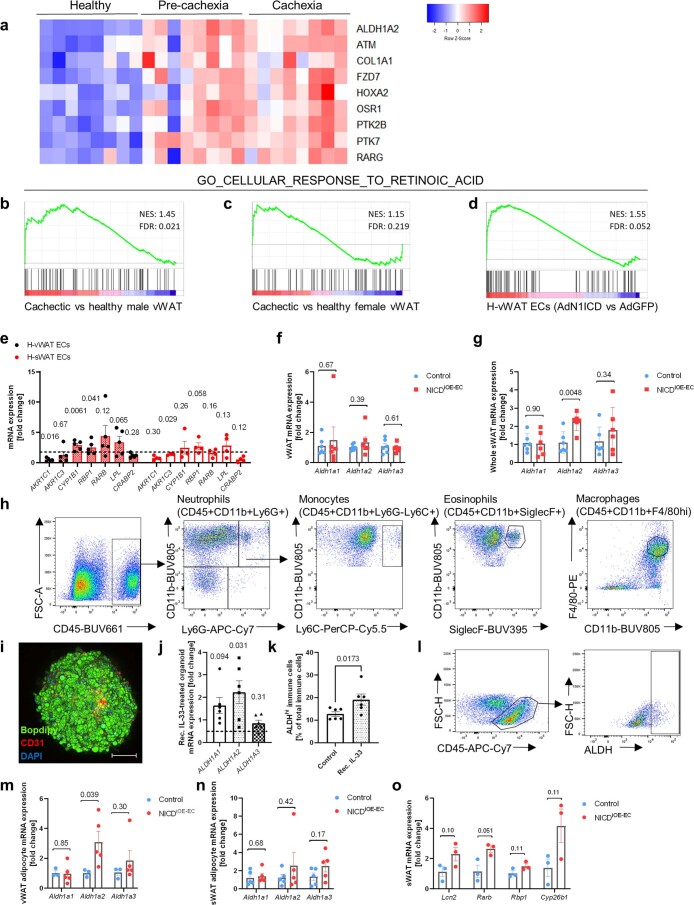
IL-33 upregulates ALDH1 and increases retinoic acid signaling in whole WAT. **a**, Heatmap of genes associated with GO term ‘cellular response to retinoic acid’ in healthy, pre-cachectic and cachectic vWAT patient samples (GSE131835). **b**–**d**, Enrichment plots of GO term ‘cellular response to retinoic acid’ in male (**b**) and female (**c**) vWAT from cachectic patients compared to healthy patient samples as well as AdN1ICD-overexpressing vWAT ECs compared to AdGFP controls (**d**). **e**, RT-qPCR analysis of genes involved in RA metabolism and/or RAR targets in human vWAT and sWAT ECs treated with AdN1ICD versus AdGFP-overexpressing cells (n = 3-5 biologically independent experiments). **f**,**g**, RT-qPCR analysis of mRNA expression levels of *Aldh1* isozymes in whole vWAT (**f**) and sWAT (**g**) from NICD^iOE-EC^ mice 4-5 weeks post-recombination (n = 6 animals per group). **h**, Gating strategy to analyse ALDH activity in myeloid cells. **i**, Human WAT organoid stained with Bodipy, anti-CD31 antibodies and DAPI. Scale bar: 200 µm. **j**, RT-qPCR analysis of *ALDH1* isozymes (n = 6 biologically independent experiments), **k**, ALDH^hi^CD45^+^ immune cells in recombinant IL-33-treated organoids (10 ng/mL) compared to controls (n = 6 biologically independent experiments). **l**, Gating strategy to analyse ALDH activity in CD45^+^ immune cells of human WAT organoids. **m**,**n**, RT-qPCR analysis of *Aldh1* isozymes in NICD^iOE-EC^ vWAT (**m**) and sWAT (**n**) adipocytes (n = 3-5 biologically independent animals). **o**, RT-qPCR analysis of genes associated with retinoic acid signalling in NICD^iOE-EC^ sWAT (n = 3 biologically independent animals). Data shown represent mean ± SEM, unpaired two-sided t-test with Welch correction (e-g, j, m, n, o) or Mann-Whitney test (k). Source data

To investigate the molecular mechanisms through which Notch1 induces WAT wasting, we performed comparative IPA of differentially expressed genes between AT-ECs from precachectic mice and N1ICD-overexpressing AT-EC datasets and their respective controls. Tretinoin, better known as all*-trans*-RA (ATRA), was identified as one of the top predicted upstream regulators in both datasets (Fig. 3a). Moreover, aldehyde dehydrogenase-1A2 (ALDH1A2) was the most enriched leading-edge gene of the ‘AT-EC Notch1 gene signature’ in whole cachectic WAT from humans (Extended Data Fig. 1h). ALDH1A2 is a key enzyme involved in the synthesis of the vitamin A metabolite RA, a potent transcriptional regulator that binds to nuclear RA receptors (RARs)^32^. In agreement with this, GSEA of the previously mentioned database (GSE131835) revealed that the Gene Ontology (GO) term ‘cellular response to RA’ was enriched in whole WAT samples from individuals with precachexia and cachexia compared to WAT samples from healthy donors (Fig. 3b and Extended Data Fig. 6a). Interestingly, subgroup analysis revealed that samples from male individuals with cachexia were more significantly enriched than samples from females compared to healthy controls (Extended Data Fig. 6b,c). This is consistent with studies showing that regulation of RA production is sex specific^33^ and our data showing that WAT wasting occurs solely in male NICD^iOE-EC^ mice.

To determine the contribution of AT-ECs to RA metabolism, we performed GSEA on RNA-sequencing (RNA-seq) data obtained from AT-ECs expressing active Notch1. When analyzing the same GO term ‘cellular response to RA’, we found that it was significantly enriched in Notch1-induced AT-ECs (Extended Data Fig. 6d). In human AT-ECs, overactivation of Notch1 led not only to a transcriptional increase of genes involved in vitamin A conversion but also to increased levels of ATRA (Fig. 3c,d).

To evaluate whether *ALDH1A2* is a direct Notch1 transcriptional target, we overexpressed N1ICD in AT-ECs and performed chromatin immunoprecipitation with sequencing (ChIP–seq) for H3K27ac, a histone mark typically found at enhancers and promoters of active genes. We observed increased H3K27ac at the Notch target gene *HEY2* and at *ALDH1A2* after N1ICD overexpression (Fig. 3e). Several RBP-J binding motifs were detected within the genomic region characterized by increased H3K27ac after N1ICD overexpression. This was confirmed by increased ALDH1A2 mRNA and protein levels (Fig. 3f–h) and altered transcription of RAR target genes and genes that are involved in RA synthesis (Extended Data Fig. 6e). Moreover, higher levels of *Aldh1a3*, another isoform of the RA-producing ALDH1 family, were found in mouse AT-ECs (Fig. 3i), indicating species-specific differences in Notch1-regulated ALDH1 isoforms.

Analysis of ALDH1 expression in whole WAT from NICD^iOE-EC^ mice revealed that in vWAT, no significant increase was detectable at the whole-tissue level (Extended Data Fig. 6f); however, levels of both *Aldh1a2* and *Aldh1a3* isozymes were increased in sWAT (Extended Data Fig. 6g), suggesting that RA signaling is active not only in ECs but also in other WAT cell types.

### WAT loss is stimulated by RA- and IL-33-dependent mechanisms

**Fig. 4:**
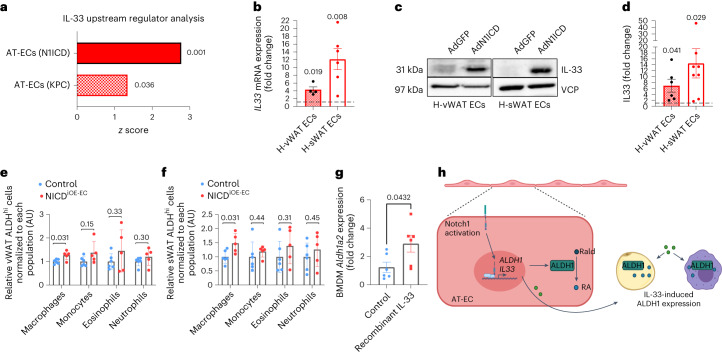
Notch1-induced IL-33 secretion increases whole-tissue ALDH1. **a**, IPA comparative analysis of N1ICD-overexpressing AT-ECs and precachectic KPC AT-ECs identified IL-33 as a potential upstream regulator of transcriptomic changes. **b**, *IL33* mRNA levels in N1ICD- compared to GFP-overexpressing human AT-ECs (*n* = 4–6 biologically independent experiments). **c**,**d**, Western blots (**c**) of human AT-EC IL-33 protein levels and quantification (**d**). Data were normalized to the expression of VCP (*n* = 6–8 biologically independent experiments). **e**,**f**, Analysis of Aldefluor activity in myeloid cells, including macrophages (CD45^+^CD11b^+^F4/80^hi^), monocytes (CD45^+^CD11b^+^Ly6G^–^Ly6C^+^), eosinophils (CD45^+^CD11b^+^SiglecF^+^) and neutrophils (CD45^+^CD11b^+^Ly6G^+^), by flow cytometry in vWAT (**e**) and sWAT (**f**) of NICD^iOE-EC^ mice (*n* = 5–6 animals per group). Quantifications were normalized to each respective cell population. Flow cytometry experiments in **e** and **f** analyzing ALDH^hi^ macrophages were performed twice with consistent results. Immunostainings and gatings for other ALDH^hi^ myeloid cell populations were performed once. **g**, RT–qPCR analysis of *Aldh1a2* mRNA expression in BMDMs treated with recombinant IL-33 for 72 h (*n* = 6 biologically independent experiments). **h**, Summary. WAT endothelial Notch1 mediates whole-tissue ALDH1 expression and RA production both directly and indirectly (via IL-33). Rald, Retinaldehyde. Data shown represent mean ± s.e.m. and were analyzed by unpaired, two-sided *t*-test with Welch correction. Source data

Upstream regulator analysis predicted the alarmin IL-33 as a potential upstream regulator in both AT-ECs with overactive Notch1 signaling and KPC AT-ECs (Fig. 4a). IL-33 is a bona fide endothelial Notch1 target in human umbilical vein ECs^34^, which we could confirm in AT-ECs (Fig. 4b–d). IL-33 regulates adipose tissue beiging through regulation of type 2 immune responses^26^. Both processes occur in NICD^iOE-EC^ mice (see earlier) and cancer cachexia models^26^. Interestingly, IL-33 has also been shown to induce ALDH1A2 expression in pancreatic myeloid cells^35^.

To investigate the potential contribution of IL-33 to RA production, ALDH activity was analyzed in myeloid cells from NICD^iOE-EC^ mice (Extended Data Fig. 6h). Only macrophages (CD45^+^CD11b^+^F4/80^hi^) showed increased ALDH activity (Fig. 4e,f), and recombinant IL-33 directly increased *Aldh1a2* expression in bone marrow-derived macrophages (BMDMs; Fig. 4g). Treatment with recombinant IL-33 induced *ALDH1A2* mRNA expression in human WAT organoids differentiated from stromal vascular fraction (SVF) cells and increased ALDH activity in CD45^+^ immune cells (Extended Data Fig. 6i–l), thus confirming that this mechanism is not restricted to mouse cells. Mature adipocytes from NICD^iOE-EC^ mice also exhibited increased *Aldh1a2* expression (Extended Data Fig. 6m,n). This led to an increase in whole-tissue expression of RAR target genes (Extended Data Fig. 6o), suggesting that enhanced ALDH1 is not limited to ECs. Taken together, endothelial Notch1 induces ALDH1-mediated RA synthesis in the endothelium and further potentiates whole-tissue RA production through angiocrine-mediated IL-33 signaling by acting on macrophages and adipocytes (Fig. 4h).

### RA regulates proapoptotic IGFBP3 in AT-ECs

**Extended Data Fig. 7:**
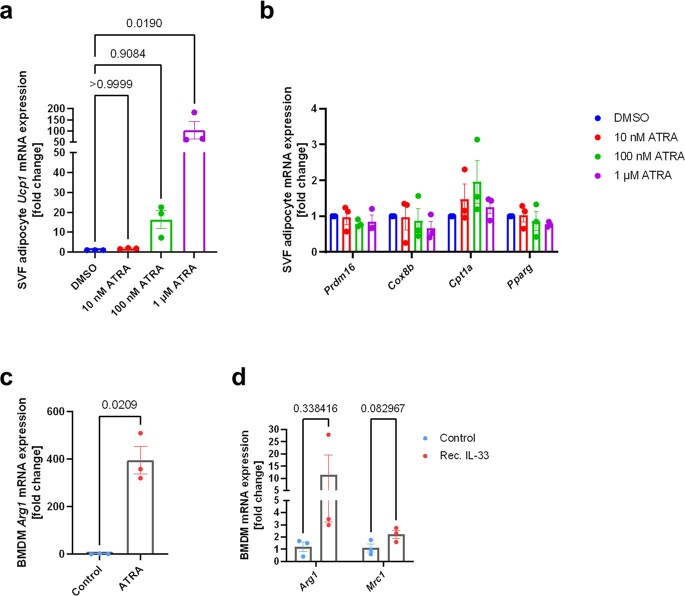
Retinoic acid signaling in adipocytes and macrophages. **a**,**b**, RT-qPCR analysis of *Ucp1* (**a**) and beiging markers (**b**) in SVF-differentiated adipocytes treated with 0 nM (DMSO only), 10 nM, 100 nM or 1 µM ATRA for 24 hours (n = 3 biologically independent experiments). **c**, RT-qPCR analysis of *Arg1* in BMDMs treated with ATRA for 72 hours (n = 3 biologically independent experiments). **d**, RT-qPCR analysis of *Arg1* and *Mrc1* in BMDMs treated with recombinant IL-33 for 72 hours (n = 3 biologically independent experiments). Data shown represent mean ± SEM, 1-way ANOVA (a), 2-way ANOVA (b) or unpaired two-sided t-test with Welch correction (c,d). Source data

Next, we asked how enhanced RA production could be causative for WAT wasting. Both RA and IL-33 can act in a paracrine manner and are regulators of thermogenesis^26,36,37^. *Ucp1* is a direct RAR transcriptional target^38^, while IL-33 induces a type 2 immune response, which promotes thermogenesis and is required for cold-induced WAT beiging in adult mice^26^. We could confirm that ATRA stimulates *Ucp1* expression in a dose-dependent manner without any effect on other beiging markers (Extended Data Fig. 7a,b). Furthermore, ATRA and recombinant IL-33 both invoked gene expression of *Arg1* in BMDMs (Extended Data Fig. 7c,d), which is similar to previous reports^39,40^.

**Fig. 5:**
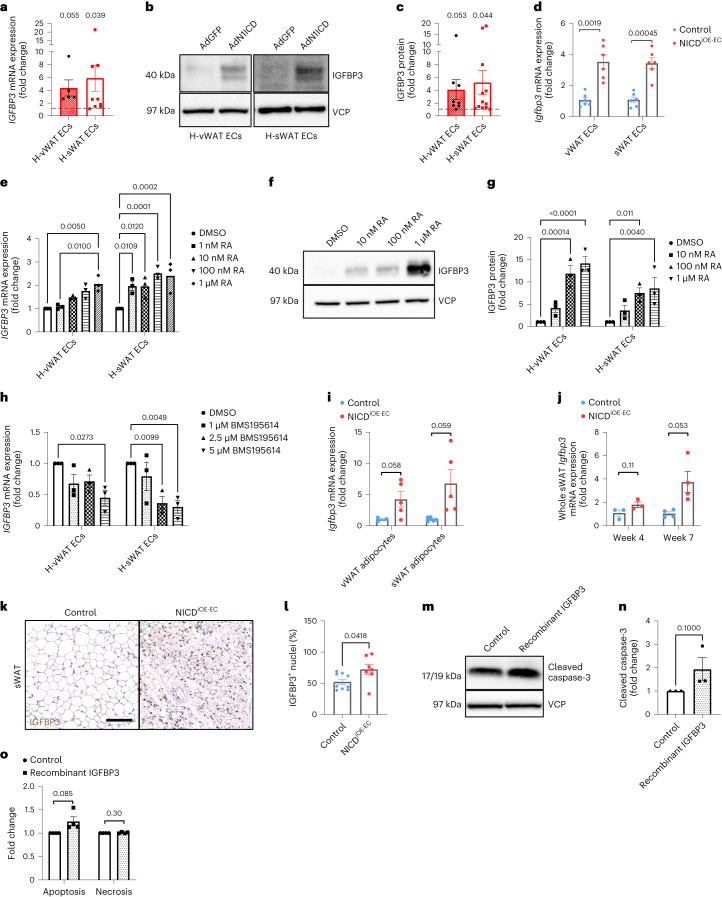
RA-regulated IGFBP3 production induces WAT apoptosis. **a**, RT–qPCR analysis of *IGFBP3* mRNA levels in AdN1ICD-overexpressing human AT-ECs compared to AdGFP controls (*n* = 5 (vWAT ECs) or 9 (vWAT ECs) biologically independent experiments). **b**,**c**, Representative western blot (**b**) and quantification (**c**) of IGFBP3 protein levels normalized to VCP (*n* = 8 (vWAT ECs) or 12 (sWAT ECs) biologically independent experiments). **d**, *Igfbp3* mRNA levels in NICD^iOE-EC^ AT-ECs isolated from male mice at 2 weeks after tamoxifen treatment (*n* = 6 animals per group). **e**,**f**, RT–qPCR (**e**) and western blotting (**f**) of IGFBP3 in human AT-ECs treated with 0 nM (DMSO only), 10 nM, 100 nM or 1 µM ATRA. The western blot image is representative of three individual experiments; *n* = 3 biologically independent experiments. **g**, IGFBP3 protein levels were quantified relative to VCP (*n* = 3 biologically independent experiments). **h**, RT–qPCR analysis of *IGFBP3* expression in human vWAT and sWAT ECs after treatment with 1, 2.5 or 5 µM RAR antagonist BMS195614 or DMSO (*n* = 3 biologically independent experiments). **i**,**j**, RT–qPCR analysis of *Igfbp3* levels in isolated NICD^iOE-EC^ vWAT and sWAT adipocytes (*n* = 3–5 animals per group; **i**) as well as whole sWAT (**j**) at 4 and 7 weeks after recombination (*n* = 3 (week 4) or 4 (week 7) animals per group). **k**, IGFBP3 (DAB) immunohistochemical stainings of sWAT from NICD^iOE-EC^ mice at 7 weeks after recombination; scale bar, 100 µm. Images are representative of two individual experiments. **l**, IGFBP3^+^ nuclei were quantified as a percentage of total nuclei (*n* = 7–9 animals per group pooled from two independent cohorts). **m**,**n**, Western blot (**m**) and quantification (**n**) of cleaved caspase-3 levels of SVF-differentiated adipocytes treated with recombinant IGFBP3 (100 ng ml^–1^) for 72 h. Data were normalized to VCP (*n* = 3 biologically independent experiments). **o**, Apoptosis (PS) and necrosis (7-AAD) were assessed using the Apoptosis/Necrosis Assay kit from Abcam (*n* = 4 biologically independent experiments; shown are biological replicates representing the averages of five technical replicates). Data shown represent mean ± s.e.m. and were analyzed by unpaired, two-sided *t*-test with Welch correction (**a**, **c**, **d**, **i** and **j**), two-way analysis of variance (ANOVA) with Dunnett’s test (**e**, **g** and **h**), Mann–Whitney test (**l** and **n**) or Sidak’s multiple comparisons test (**o**). The experiment in **d** was performed twice with consistent results. Shown is one representative experiment. Experiments in **k** and **l** were performed in two independent cohorts, and results were pooled. Results were consistent between the two experiments. Source data

**Extended Data Fig. 8:**
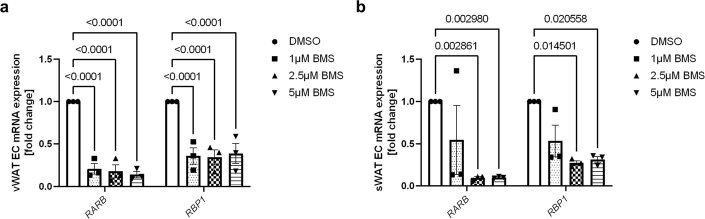
BMS195614 downregulates RAR target genes in AT-ECs *in vitro*. **a**,**b**, RT-qPCR analysis of prototypical RAR target genes in human vWAT (**a**) and sWAT (**b**) ECs upon treatment with 1, 2.5 or 5 µM of RAR antagonist BMS195614 or DMSO (n = 3 biologically independent experiments). Data shown represent mean ± SEM, 2-way ANOVA with Dunnett’s test (a, b). Source data

Cachectic WAT loss is also mediated through adipocyte apoptosis and impaired adipogenesis^5^. *IGFBP3* is a RARα target gene^41^ that inhibits adipogenesis and induces apoptosis in several cell types^42^. We observed IGFBP3 upregulation in human and mouse AT-ECs after Notch1 overactivation (Fig. 5a–d). Dose-dependent increases in IGFBP3 were also detected in human AT-ECs treated with ATRA (Fig. 5e–g), whereas inhibition of RAR-mediated transcription using BMS195614, a RARα antagonist, inhibited both classical RAR target genes and *IGFBP3* expression (Fig. 5h and Extended Data Fig. 8a,b). Consistent with the observation that EC Notch1 induces *Aldh1a2* expression in adjacent cell types (Fig. 4 and Extended Data Fig. 6f–o), adipocytes and whole sWAT of NICD^iOE-EC^ mice also demonstrated increased *Igfbp3* expression (Fig. 5i–l).

To test the effect on WAT adipogenesis and apoptosis, SVFs from wild-type mice were differentiated into adipocytes and treated with recombinant IGFBP3. IGFBP3 is known to inhibit the expression of adipogenic markers during adipocyte differentiation of 3T3-L1 cells^42^. Notably, IGFBP3 also induced cleavage of caspase-3 and apoptosis (Fig. 5m–o). Taken together, the data reveal that endothelial Notch1 drives RA-induced expression of the thermogenic protein UCP1, a type 2 immune response and apoptosis in WAT.

### RA metabolism is overactive during precachexia

**Fig. 6:**
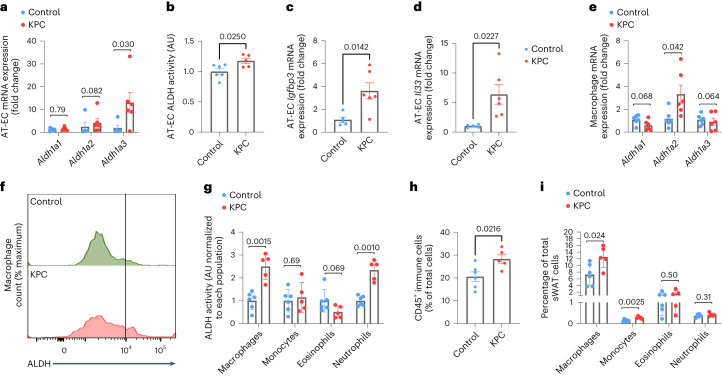
Notch1-driven changes in vitamin A metabolism occur during precachexia. **a**, RT–qPCR analysis of *Aldh1* mRNA levels in AT-ECs from KPC mice (*n* = 5–6 animals per group). **b**, AT-EC Aldefluor activity in KPC mice versus in non-tumor-bearing control mice (*n* = 5–6 animals per group). **c**, RT–qPCR analysis of *Igfbp3* mRNA levels in AT-ECs (*n* = 5–6 animals per group). **d**, RT–qPCR analysis of *Il33* mRNA levels in AT-ECs (CD31^+^CD45^–^) from KPC and control mice (*n* = 6 animals per group). **e**, *Aldh1a2* mRNA expression in macrophages (CD45^+^CD11b^+^F4/80^hi^) from KPC mice (*n* = 6 animals per group). **f**, Flow cytometry histogram plot of Aldefluor activity in control and KPC macrophages. **g**, Analysis of ALDH activity in myeloid cells, including macrophages (CD45^+^CD11b^+^F4/80^hi^), monocytes (CD45^+^CD11b^+^Ly6C^+^), eosinophils (CD45^+^CD11b^+^SiglecF^+^) and neutrophils (CD45^+^CD11b^+^Ly6G^+^), measured by flow cytometry in sWAT from KPC and control mice (*n* = 5–6 animals per group). **h**,**i**, CD45^+^ immune cells (**h**) and myeloid cell populations (**i**) quantified as a percentage of total living cells (DAPI^–^) in KPC and control sWAT (*n* = 5–6 animals per group). Data shown represent mean ± s.e.m. and were analyzed by Mann–Whitney test (**a**) or unpaired, two-sided *t*-test with Welch correction (**b**–**e** and **g**–**i**). Experiments in **a**, **c** and **d** were performed twice with consistent results. Shown are data from one representative experiment. Source data

**Extended Data Fig. 9:**
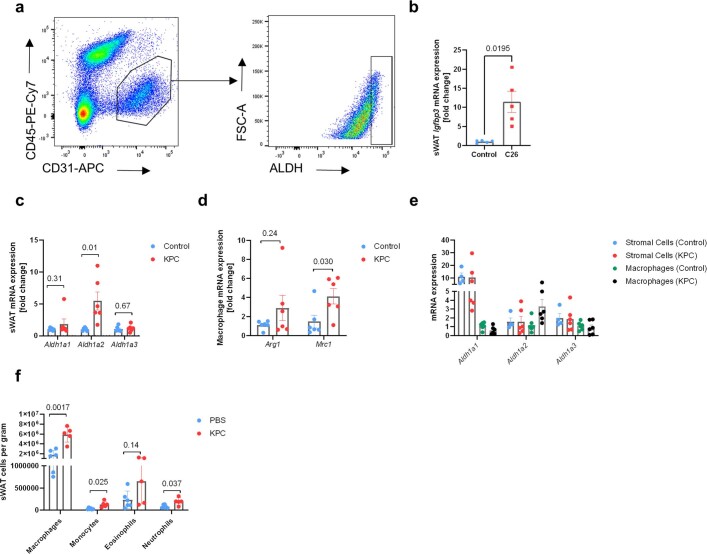
Confirmation of NICD^iOE-EC^ findings in C26 and KPC cachexia models. **a**, Gating strategy to quantify the percentage of ALDH^hi^ AT-ECs of KPC pre-cachectic male mice. **b**, RT-qPCR analysis of *Igfbp3* in whole sWAT from C26 cachectic mice compared to non-tumour bearing controls (n = 5 animals per group). **c**, RT-qPCR analysis of *Aldh1* isozymes in whole sWAT from KPC pre-cachectic mice compared to non-tumour bearing controls (n = 6 animals per group). **d**, RT-qPCR analysis of *Arg1* and *Mrc1* in sorted macrophages (CD45^+^CD11b^+^F4/80^hi^) from KPC and non-tumour bearing mice (n = 6 animals per group). **e**, RT-qPCR analysis and comparison of *Aldh1* isozymes in sWAT stromal cells (CD140a^+^Sca1^+^CD45^−^CD31^−^) vs. macrophages (CD45^+^CD11b^+^F4/80^hi^) FACS-sorted from KPC pre-cachectic mice compared to non-tumour bearing controls (n = 6 animals per group). **f**, Flow cytometry analysis of the number of myeloid cells per gram of sWAT, including macrophages (CD45^+^CD11b^+^F4/80^hi^), monocytes (CD45^+^CD11b^+^Ly6C^+^), eosinophils (CD45^+^CD11b^+^SiglecF^+^) and neutrophils (CD45^+^CD11b^+^Ly6G^+^), from pre-cachectic KPC mice (n = 5–6 animals per group). Data shown represent mean ± SEM, unpaired two-sided t-test with Welch correction (b, c, d, f). Source data

As many data were obtained in Notch1 gain-of-function models, we aimed at investigating whether changes in ALDH1, IL-33 and IGFBP3 expression are present in WAT of mice bearing KPC tumors during precachexia. Indeed, *Aldh1a3* expression and ALDH enzyme activity were elevated in AT-ECs of tumor-bearing mice compared to in tumor-free wild-type mice (Fig. 6a,b and Extended Data Fig. 9a). This was accompanied by increased expression of the RA target gene *Igfbp3* (Fig. 6c). Additionally, a stark increase in *Igfbp3* mRNA could be observed in whole sWAT from C26 cachectic mice (Extended Data Fig. 9b).

Mouse AT-ECs express low IL-33 levels in a basal state^43^; however, in the context of inflammation and disease, ECs strongly increase their IL-33 production^44^. Again, we detected increased *Il33* expression in AT-ECs of tumor-bearing wild-type mice (Fig. 6d). This was associated with increased *Aldh1a2* expression at the whole-tissue level and in macrophages along with type 2 inflammatory markers (Fig. 6e and Extended Data Fig. 9c,d). *Aldh1a2* expression was found to be comparable in stromal cells and macrophages, confirming that, despite the high expression of ALDH1 by stromal cells, macrophages are also major contributors to whole WAT RA production (Extended Data Fig. 9e). Higher ALDH activity in macrophages and neutrophils was also observed as well as increased numbers of macrophages and monocytes in WAT (Fig. 6f–i and Extended Data Fig. 9f). As such, the molecular mechanisms occurring over the course of WAT wasting in NICD^iOE-EC^ mice, as described above, could also be detected in WAT in a classical cancer cachexia mouse model.

### Pharmacological inhibition of RA blocks WAT wasting

**Fig. 7:**
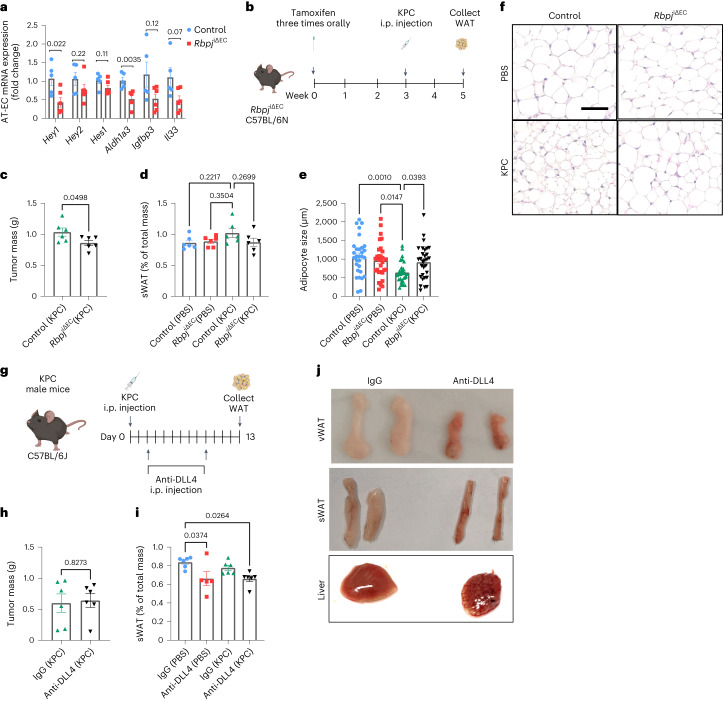
Pharmacological targeting of DLL4 does not inhibit cachexia progression. **a**, RT–qPCR analysis of AT-EC Notch1 target genes from *Rbpj*^iΔEC^ and control mice (*n* = 5–6 animals per group). **b**, Analysis scheme of *Rbpj*^iΔEC^ mice. Mice were given tamoxifen 3 weeks before injection of KPC tumor cells or PBS as a control. Samples were analyzed on day 13 after KPC injection. **c**, KPC tumor mass in *Rbpj*^iΔEC^ mice compared to in control mice (*n* = 6 animals per group). **d**, Relative sWAT mass normalized to total mass in PBS- and KPC-treated mice (n = 6 animals per group). **e**,**f**, Average adipocyte size (**e**) quantified from hematoxylin and eosin (H&E) staining (**f**) of *Rbpj*^iΔEC^ sWAT. Shown are representative images from all groups; scale bar, 50 µm (*n* = 6 animals per group, five images per mouse). **g**, Analysis scheme of DLL4-neutralizing antibody treatment in KPC mice (*n* = 5–6 animals per group). **h**, KPC tumor mass in wild-type C57BL/6J mice treated with control IgG or anti-DLL4 (*n* = 6 animals per group). **i**, Relative sWAT mass normalized to total mass (*n* = 5–6 animals per group). **j**, Representative images of vWAT, sWAT and liver from non-tumor-bearing control mice injected with IgG or anti-DLL4. Data shown represent mean ± s.e.m. and were analyzed by unpaired, two-sided *t*-test with Welch correction (**a**, **c** and **h**) or one-way ANOVA with Tukey’s test (**d**, **e** and **i**). Source data

Last, we aimed at blocking angiocrine signaling to inhibit WAT wasting. First, we used the *Cdh5-cre*^ERT2^; *Rbpj*^*lox*/*lox*^ (*Rbpj*^iΔEC^) Notch loss-of-function mouse model to delete the *Rbpj* gene encoding the Notch nuclear transducer RBP-Jκ in ECs of adult mice^19,25^. In AT-ECs, expression of prototypical Notch target genes and *Aldh1a3*, *Il33* and *Igfbp3* were reduced (Fig. 7a), reaffirming that the genes identified are Notch1 targets. As expected^45^, growth of a solid tumor mass (KPC) was reduced in *Rbpj*^iΔEC^ mice (Fig. 7b,c), thus precluding meaningful conclusions about WAT wasting. Nevertheless, tumor-induced reductions in adipocyte size were prevented in *Rbpj*^iΔEC^ mice (Fig. 7d–f).

Next, we verified whether neutralization of the Notch1 ligand DLL4 could serve as a therapeutic treatment. Treatment with DLL4-neutralizing antibodies (Fig. 7g) had little effect on primary tumor growth at this rather early time point (Fig. 7h) but reduced WAT mass and induced a brown-like appearance (Fig. 7i,j), likely from Notch activity inhibition directly in adipocytes, as previously described^46^. In addition, treatment with anti-DLL4 caused vascular malformations in the liver (Fig. 7j), as expected^47^.

**Fig. 8:**
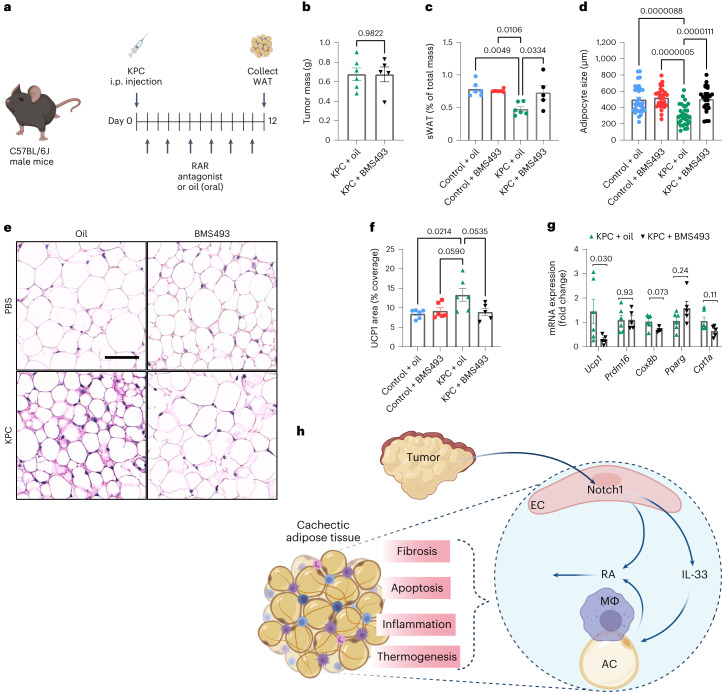
Pharmacological blockade of RA signaling inhibits WAT remodeling in cachexia. **a**, Experimental setup of treatments given to KPC or non-tumor-bearing control mice. Mice were given BMS493 (RAR antagonist) or a solvent control orally every second day following KPC or PBS injection. **b**, Tumor mass of KPC mice given either oil or BMS493 (*n* = 5–6 animals per group). **c**, Percentage of sWAT mass normalized to total body mass (*n* = 5–6 animals per group). **d**, Adipocyte size quantified from sWAT H&E stainings (*n* = 5–6 animals per group, five images per mouse). **e**, Representative images of H&E-stained sWAT from one experiment; scale bar, 50 µm. **f**, UCP1 (DAB) immunohistological stainings were quantified as a percentage of total coverage area (*n* = 5–6 biologically independent animals). **g**, RT–qPCR analysis of thermogenic and/or beiging markers in whole sWAT tissue (*n* = 5–6 animals per group). **h**, Summary. Tumor-induced WAT endothelial Notch1 signaling mediates various hallmarks of adipose tissue wasting; MΦ, macrophage. Data shown represent mean ± s.e.m. and were analyzed by unpaired, two-sided *t*-test with Welch correction (**b**), one-way ANOVA with Tukey’s test (**c**, **d** and **f**) or Mann–Whitney test (**g**). Source data

**Extended Data Fig. 10:**
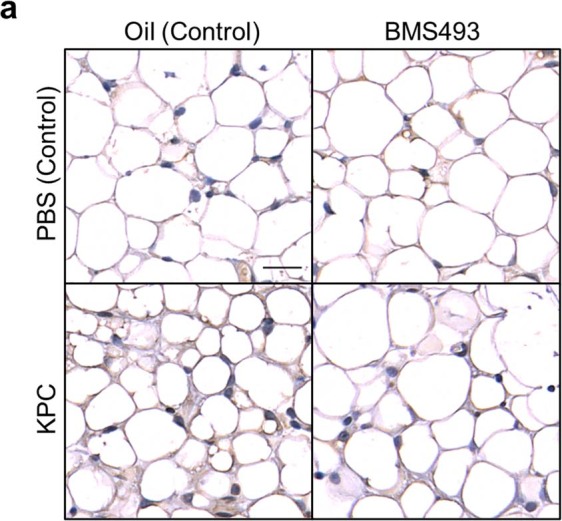
UCP1 immunohistochemical stainings of sWAT from KPC mice treated with BMS493 or DMSO (solvent control). **a**, Representative images of UCP1 (DAB) stainings of sWAT from KPC or non-tumour bearing mice which received either oil (solvent control) or BM493 treatment orally from one individual experiment. Scale bar: 100 µm.

Because targeting of Notch led to severe side effects, we aimed at specifically targeting RA downstream of Notch to prevent WAT loss in the mouse KPC cachexia model (Fig. 8a). Tumor-bearing mice were treated with the pan-RAR oral antagonist BMS493 or solvent control. BMS493-treated mice showed no difference in tumor mass compared to controls (Fig. 8b); however, BMS493 prevented WAT loss and changes in adipocyte size (Fig. 8c–e). This was associated with reduced expression of UCP1 (Fig. 8f,g and Extended Data Fig. 10a). As such, this well-tolerated treatment inhibited WAT wasting in a pancreatic cancer cachexia model without affecting growth of the primary tumor mass.

In summary, the data suggest that tumor-induced Notch signaling activation in AT-ECs leads to excessive RA production in WAT, which mediates all hallmarks of WAT wasting in an angiocrine manner (Fig. 8h).

## Discussion

Tumor-secreted factors change gene transcription in the endothelium in organs at distant sites to favor a prometastatic microenvironment^11,12^. Similarly, this study shows that WAT endothelial gene transcription already becomes modified during precachexia and that this subsequently augments tumor-induced WAT loss. As such, one can assume that the endothelium amplifies cancer-secreted factors, putting angiocrine signaling into a central position orchestrating both metastasis and cachexia.

This work identified Notch1 signaling as a major regulator of cancer-induced WAT remodeling. Future studies will have to dissect the exact mechanism of how tumors overactivate Notch signaling in ECs at distant sites, particularly in humans. Here, we show that the prototypical cachexokines^6,7^ TNF-α and IL-1β can induce expression of the Notch ligand JAG1. Others have previously shown that these proinflammatory cytokines induce JAG1 expression in ECs of other tissues^16^. Additionally, tumor-released exosomes may also transfer DLL4 to activate Notch receptors on ECs at distant sites^48,49^, so there are several possibilities of how tumors change the endothelial transcriptome across large distances in the body. Notably, a moderate but sustained increase in EC Notch activity was (even in the absence of a tumor) already sufficient to induce WAT wasting characterized by increased apoptosis, impaired preadipocyte differentiation, increased myeloid cell infiltration and inflammation, increased UCP1 expression with beiging, impaired lipid storage and eventually fibrosis. These are the hallmarks of WAT remodeling in cancer cachexia, which cause severely impaired organ function^1,3,4,6,30,50^.

It is well known that although females are less susceptible to cachexia development than males, the underlying mechanisms remain speculative^24^. Interestingly, female NICD^iOE-EC^ mice did not develop signs of WAT remodeling in the time course of our study. This observation aligns with previously known sex-specific differences in adipose tissue ALDH1 expression^33,51^ and fits perfectly with the sex-specific enrichment of the GO term ‘cellular response to RA signaling’ observed in adipose tissue from individuals with cachexia. It will be of outstanding interest to unravel the responsible mechanism in future studies.

Mechanistically, sustained endothelial Notch1 signaling increased RA and IL-33 synthesis in ECs. However, ECs were not the only cell type producing RA in WAT in humans and in mouse cancer models and NICD^iOE-EC^ mice. The angiocrine factor IL-33, a well-known target of Notch signaling in ECs^34^, induces excessive RA production in neighboring adipocytes and macrophages. This activates a macrophage phenotype that helps sustain catecholamine-induced WAT loss^30^. RA also increased the expression of antiadipogenic and proapoptotic IGFBP3, thus impacting lipid storage and adipocyte turnover^42^. This appears to be a highly conserved mechanism as ImpL2, an IGFBP homolog in *Drosophila*, also mediates systemic wasting^20,21^.

This study suggests that Notch and RA signaling play a major role in mediating WAT remodeling in cancer cachexia. From a translational point of view, blocking these pathways would be highly desirable. However, in line with previous reports^45^, Notch inhibition had severe side effects, questioning the use of Notch-inhibiting drugs in humans at least when given over long time periods^25,52^. Targeting RA signaling downstream of Notch using an oral pan-RAR antagonist inhibited WAT wasting in a pancreatic cancer cachexia model without inducing Notch-dependent side effects. As RA signaling is crucial for many biological functions, further research is needed to determine whether long-term intake of such drugs would be tolerable.

Taken together, this study showed that the large surface of the adipose tissue endothelium is capable of mediating WAT wasting in an angiocrine manner, where Notch1 and downstream RA signaling play crucial roles and RA signaling might serve as a targetable pathway to inhibit the course of tissue wasting.

## Methods

### Animal experiments

All animal procedures were performed in accordance with institutional and national regulations and were approved by local committees for animal experimentation (RP Karlsruhe, German Cancer Research Center (DKFZ), Heidelberg University and RP Upper Bavaria). Animals were housed under specific pathogen-free barrier conditions and were fed a standard mouse chow ad libitum (3437, Granovit). Animals were housed at 22 ± 2 °C with 60% humidity and a 12-h light/12-h dark rhythm. Tumor experiments using wild-type mice were performed using 10‐ to 14‐week‐old male BALB/c mice obtained from Charles River Laboratories. *Rbpj*^iΔEC^ mice (C57BL/6N), NICD^iOE-EC^ mice (C57BL/6J) and wild-type C57BL/6J mice were bred in-house at the DKFZ under specific pathogen-free conditions. Animals included in tumor experiments were extensively assessed daily based on score sheets with criteria including body condition scoring and physical examination to prevent animal burden. Maximal burden was not exceeded with any animal.

Sex was considered in all aspects of the study. NICD^iOE-EC^ experiments were initially performed in both sexes to analyze phenotype. A phenotype was only seen in male mice. Based on this, only male mice were used for the C26 and KPC models, as the purpose was to analyze downstream components of the Notch pathway that contribute to WAT remodeling.

#### NICD^iOE-EC^ mice

Tamoxifen-inducible, EC-specific Notch1 activation was induced in flox-Notch1-ICD mice (Jackson Laboratory) crossed with *Cdh5*(PAC)*cre*^ERT2^ mice^18,19^ (C57BL/6J background). Tamoxifen dissolved in olive oil was administered either i.p. (1.5 mg for 5 consecutive days) or dissolved in peanut oil and administered orally (2 mg for 3 consecutive days) at 8–12 weeks of age. Control mice that did not express Cre^ERT2^ were also treated with tamoxifen. We have previously reported extensive phenotyping of NICD^iOE-EC^ mice treated under the same conditions^17,21^.

#### *Rbpj*^iΔEC^ mice

Tamoxifen-inducible, EC-specific *Rbpj* deletion was induced in *Rbpj*^*lox*/*lox*^ mice (Jackson Laboratory) crossed with *Cdh5*(PAC)*cre*^ERT2^ mice^19,25^ (C57BL/6N background). Tamoxifen dissolved in peanut oil was administered orally (2 mg for 3 consecutive days) at 9–13 weeks of age. Control mice that did not express Cre^ERT2^ were also treated with tamoxifen. *Rbpj*^iΔEC^ mice were injected with PBS or KPC cells at 3 weeks following oral administration of tamoxifen.

#### KPC model

Mice were injected with cells from the pancreatic ductal adenocarcinoma cell line derived from KPC mice (*Kras*^G12D^; *Trp53*^R172H^; *Elas*-*cre*^ER^)^53^. KPC cells (10^6^) in 100 μl PBS were injected i.p. into 9- to 14-week-old C57BL/6J or *Rbpj*^iΔEC^ mice and compared to PBS-injected, age-matched littermate controls. Tumor growth and animal well-being were closely inspected daily according to score sheets monitoring variables such as body weight, behavior and tumor growth (by palpations). To analyze gene expression during precachexia (<10% change in body mass), mice were analyzed at 11 d after tumor injection.

#### RAR antagonist treatment (KPC model)

BMS493 (5 mg per kg (body weight) dissolved in peanut oil; Sigma-Aldrich) or peanut oil was administered to wild-type C57BL/6J mice every second day (1, 3, 5, 7, 9 and 11 d) following KPC or PBS i.p. injection (day 0). Mice were analyzed at 12 d after KPC or PBS injection at a precachectic time point (<10% change in body mass).

#### Anti-DLL4 treatment (KPC model)

DLL4-neutralizing antibodies from Genentech (10 mg per kg (body weight) in PBS) or control IgG (JIM-009-000-003, Biozol Diagnostica; 10 mg per kg (body weight) in PBS) were administered i.p. to C57BL/6J mice 2 and 9 d after KPC or PBS i.p. injection (day 0). Mice were analyzed at 13 d after KPC or PBS injection at a precachectic time point (<10% change in body mass).

#### C26 cachexia model

C26 cells (10^6^) were injected subcutaneously into BALB/c mice. Non‐tumor-bearing control mice were injected with 50 μl of Dulbecco’s PBS. Mice were analyzed 16–21 d after tumor cell injection at a cachectic time point (>10% loss in body mass).

### Plasma collection and measurement of metabolites and proteins

Blood was collected following anesthetization (100 mg per kg (body weight) ketamine and 14 mg per kg (body weight) xylazine) and by cardiac puncture before cervical dislocation. Plasma leptin was quantified using the Proteome Profiler Adipokine Array kit (R&D Systems). TAG and glycerol (Sigma), NEFAs (Wako Chemicals) and very low-density lipoprotein/low-density lipoprotein and high-density lipoprotein levels (Sigma) were measured using kits according to manufacturer’s instructions. Blood was collected from the tail vein at approximately 0800–0900 h without fasting. Basal blood glucose levels were measured using the Free-Style Lite blood sugar measuring system.

### scioCyto cytokine array of serum

Blood was taken from the vena facialis 11 d after tumor injection and collected in a microvette LH (Sarstedt, 201.345) to receive serum. The company Sciomics was running the scioCyto Cytokine Array to compare relative protein levels in serum from tumor-bearing mice to those from control mice. Acquired raw data were analyzed using the linear models for microarray data (LIMMA) package of R/Bioconductor after uploading the median signal intensities. For normalization, a specialized invariant Lowess method was applied. For analysis of the samples, a one-factorial linear model was fitted with LIMMA, resulting in a two-sided *t*-test or *F*-test based on moderated statistics. All presented *P* values were adjusted for multiple testing by controling the false discovery rate according to Benjamini and Hochberg.

### Cell lines

HEK293A cells were obtained as part of the ViraPower Adenoviral Expression System (Thermo Fisher) and used for adenovirus production. The KPC cell line was a gift from S. Konieczny (Purdue University). The *Drosophila melanogaster* Schneider cell line was a gift from R. Renkawitz and M. Bartkuhn (University of Giessen).

### AT-EC isolation for cell culture

ECs were isolated from human visceral abdominal and subcutaneous abdominal adipose tissue biopsies and collected from individuals undergoing bariatric surgery at the University Hospital Heidelberg. Collection was approved by the Institutional Review Board of the Medical Faculty of the University of Heidelberg in accordance with the Declaration of Helsinki. All individuals gave preoperative consent. Adipose tissue depots were minced and digested in 2 mg ml^–1^ collagenase II (Thermo Fisher Scientific) and 2 mg ml^–1^ dispase II (Pan Biotech) in Dulbecco’s PBS. Homogenates were filtered through a 100-µm cell strainer and diluted 1:1 with PBS. SVF pellets centrifuged at 300*g* for 5 min were suspended in 3 ml of PBS + 10% fetal calf serum (FCS). Twenty microliters of CD31-Dynabeads (Thermo Fisher Scientific) per depot was washed three times in 5 ml of PBS + 10% FCS and diluted in 500 µl of PBS containing 10% FCS per depot. Five hundred microliters of bead suspension was added to cells and suspended in 15-ml tubes. Tubes were incubated on a rotator at room temperature for 45 min. Cells were placed on a magnetic rack and washed three times with PBS containing 10% FCS. Cells were suspended in EGM-2 medium (Lonza) and seeded into flasks or six-well plates precoated with 0.5% gelatin. Cells were cultured for up to five passages using trypsin-EDTA (0.05%; Thermo Fisher Scientific).

AT-ECs were treated with adenoviral supernatants at optimized titers for 24 h and washed twice with PBS, and medium was replaced with fresh EGM-2 medium (Lonza). Cells were collected for protein or RNA lysates after 24 h in fresh medium. AT-ECs treated with ATRA (1–1,000 nM; Cayman Chemical) or RAR antagonist BMS195614 (1–5 µM; Tocris Bioscience) were incubated for 48 h before RNA or protein isolation.

### Mouse SVF isolation and differentiation

Adipose tissue samples from wildtype C57BL/6J mice were digested and centrifuged as described above. SVFs were resuspended in ACK lysis buffer (Thermo Fisher Scientific) for 1 min before dilution with 10 ml of PBS. Cells were centrifuged at 300*g* for 5 min and suspended in high-glucose DMEM containing GlutaMAX (Thermo Fisher Scientific) + 10% FCS and seeded in six-well plates. To induce adipocyte differentiation, medium was changed to high-glucose DMEM containing GlutaMAX with 20% FCS, 1% penicillin/streptomycin (P/S), 0.5 mM 3-isobutyl-1-methylxanthin (Cayman Chemical), 5 µg ml^–1^ insulin (Sigma-Aldrich), 4 µM rosiglitazone (Cayman Chemical) and 10 mM dexamethasone (Cayman Chemical) for 4 d. Medium was exchanged for 2 d to high-glucose DMEM containing GlutaMAX supplemented with 20% FCS, 1% P/S and 5 µg ml^–1^ insulin, followed by another 3 d in fresh medium. Medium was replaced with basal high-glucose DMEM containing GlutaMAX with 1% P/S for experimentation. SVFs were treated before or after differentiation with recombinant mouse IGFBP3 (Merck, 100 ng ml^–1^) for 72 h in basal DMEM or in ATRA (Cayman Chemical, 10 nM, 100 nM or 1 µM in DMSO) for 24 h in basal DMEM.

### BMDM isolation and differentiation

BMDMs were isolated from C57BL/6J mice. Femurs and tibiae were flushed with DMEM. Cells were collected, centrifuged and resuspended in DMEM (Thermo Fisher) with 10% FCS. Cells were seeded on 10-cm dishes (Corning). Macrophages were differentiated with 10 ng ml^–1^ macrophage colony-stimulating factor (Peprotech). After 7 d, cells were serum starved overnight and treated with recombinant mouse IL-33 (250 ng ml^–1^; Peprotech) or ATRA (1 µM; Cayman Chemical) for 72 h.

### Human adipose tissue organoid culture

SVF pellets were isolated from human adipose tissue biopsies as described above. SVFs were seeded at 25,000 cells per well in 100 µl of EGM-2 medium into Nunclon Sphera 96-well U-shaped plates (Thermo Fisher) and were incubated for 1 week before adipocyte differentiation (as described above). Immunofluorescence stainings were performed to verify adipocyte expansion and EC maintenance. Recombinant IL-33 (10 ng ml^–1^) was added to organoids for 6 d before RT–qPCR or flow cytometry analysis. Organoids were digested with collagenase II/dispase II in PBS for 1 h before RNA isolation or flow cytometry antibody staining.

### Western blotting

Cells and tissues in lysis buffer (Cell Signaling) containing 1 mM phenylmethylsulfonyl fluoride were boiled at 95 °C for 5 min in Laemmeli buffer and separated according to molecular weight using SDS–PAGE (Bio-Rad). Blotting was performed in a blotting chamber (Peqlab Biotechnology), and proteins were transferred onto nitrocellulose paper. Membranes were blocked in 5% skim milk in TBS and 0.1% Tween 20 (TBST). Primary antibodies diluted in 3% bovine serum albumin (BSA) in TBST were added to membranes overnight. Antibodies and their dilutions are listed in the Reporting Summary. Secondary antibodies in 5% milk in TBST were added for 1 h before detection with ECL solution (Thermo Fisher Scientific) or for 1 min in AceGlow (VWR). Membranes were imaged by chemiluminescence using a ChemiDoc Imager (Bio-Rad). Bands were quantified using ImageLab software (Bio-Rad). Quantifications were normalized to the expression of VCP or β-actin (housekeeping proteins).

### Mass spectrometry sample preparation

Human AT-ECs were treated with GFP or N1ICD adenoviral vectors at optimized titers in EGM-2. After 24 h, supernatants were exchanged for fresh EGM-2 containing 10 µM all-*trans*-retinol (Sigma). Cells were collected after 16 h by scraping in 1 ml of methanol-HCl (0.25% (vol/vol)) and centrifuging (5,000*g*, 5 min). Pellets were frozen at −20 °C until measurement. ATRA concentrations were measured by mass spectrometry, as we have recently described^54^.

### ChIP–seq

*D. melanogaster* Schneider cells were grown in Schneider’s *Drosophila* medium (Gibco, 21720024) supplemented with 10% fetal bovine serum (Gibco, 10270-106), P/S (Gibco) and glutamine (Gibco, 25030-024). ChIP experiments were performed essentially as previously described^55^. Chromatin from *D. melanogaster* cells was used for spike-in purposes. Human chromatin was incubated with anti-H3K27ac (at a ratio of 2.5 μg of antibody to 100 μg of chromatin; Diagenode, pAb-174-050) in combination with anti-His2Av (at a ratio of 1 μg of antibody to 100 μg of chromatin; Active Motif, 61686) to immunoprecipitate the *Drosophila* chromatin. Libraries were prepared using a Diagenode MicroPlex Library Preparation kit v2 (Diagenode, C05010001) following the manufacturer’s instructions with few modifications. Libraries were purified with Agencourt AMPure XP Beads (Beckman Coulter, A63881), quantified, analyzed on a Tapestation device (Agilent) and pooled. Sequencing was performed at Novogene.

Raw FASTQ files were quality and adaptor trimmed using trimGalore v.0.6.5 (https://www.bioinformatics.babraham.ac.uk/projects/trim_galore/). Trimmed files were aligned against the human reference genome (hg19) using Hisat2 v.2.2.1 (ref. ^56^) and stored as binary alignment maps (BAM). Quality of the alignment was inspected and validated within R v.4.0.2 (http://www.r-project.org/index.html) using systemPipeR’s alignStats function^57^. PCR duplicates were removed using Picard tools (http://broadinstitute.github.io/picard/). Coverage tracks based on the processed BAM files were generated using Deeptools bamCoverage and stored as BigWig files (reads per kilobase per million (RPKM) normalized). Binding profiles were visualized within R using the R/Bioconductor^58^ package GVIZ^59^.

### RNA isolation from whole WAT and adipocytes

Flash-frozen WAT was lysed in 500 µl of Trizol reagent (Thermo Fisher Scientific). Tissues were homogenized using a Mixer Mill 301 homogenizer (Retsch) at a frequency of 1/30 s for 1 min, followed by a brief centrifugation. Adipocytes were isolated following mincing and digestion of adipose tissue for ~1 h at 37 °C (2 mg ml^–1^ collagenase and 2 mg ml^–1^ dispase II). Homogenates were strained through a 100-µm cell strainer and centrifuged (200*g*, 5 min). Floating fractions containing adipocytes were collected with a cut 1,000-µl pipette tip. Adipocytes were transferred to a 5-ml tube. PBS was added, and cells were centrifuged (200*g*, 5 min). PBS was carefully discarded using a Pasteur pipette, and washing was repeated twice. Adipocytes were collected in PBS and transferred to a 2-ml tube for centrifugation (200*g*, 5 min). RNA was isolated using a Trizol Plus RNA Purification kit (Thermo Fisher Scientific) according to the manufacturer’s instructions.

### RNA isolation from WAT ECs of KPC mice (for microarray only)

Sheep anti-rat IgG Dynabeads (Thermo Fisher Scientific) were washed (30 µl with 5 ml of PBS + 0.1% BSA) three times on a magnetic rack. Beads were suspended (5 ml of PBS + 0.1% BSA), and 15 µl of rat anti-mouse CD31 (BD Biosciences) or 15 µl of rat anti-mouse CD45 (BD Biosciences) was added. Dynabeads and antibody were incubated overnight at 4 °C on a tube rotator. sWAT was excised from mice and minced. Tissue was digested at 37 °C in a water bath, with mixing every 10–15 min (2 mg ml^–1^ collagenase II, 2 mg ml^–1^ dispase II and 1% delipidated BSA (DL-BSA) in PBS). Homogenates were filtered through a 100-µm cell strainer and diluted 1:1 with PBS. Digests were centrifuged (300*g*, 5 min). SVF pellets were suspended in 3 ml of PBS + 1% DL-BSA. CD31-Dynabeads and CD45-Dynabeads were washed three times (5 ml of PBS + 1% DL-BSA) and diluted in 500 µl. Five hundred microliters of CD45-Dynabeads was transferred to each cell suspension, and tubes were incubated on a rotator (4 °C, 20 min). Cells were placed on a magnetic rack and washed three times (PBS + 1% DL-BSA). Five hundred microliters of CD31-Dynabeads was transferred to each cell suspension, and tubes were incubated on a rotator (4 °C, 30 min). Cells were placed on a magnetic rack and washed three times (PBS + 1% DL-BSA). After washing, cells were centrifuged (100*g*, 10 min, 4 °C). Cells were immediately lysed, and RNA was isolated using an RNA Mini kit (Qiagen) for KPC AT-ECs.

### RNA isolation from muscle and heart ECs of KPC mice

Cells were isolated as described above except digestive solution contained 2 mg ml^–1^ collagenase II, 2 mg ml^–1^ dispase II and 2 µM CaCl_2_, and ECs were isolated by CD31^+^ selection alone.

### Flow cytometry and fluorescence-activated cell sorting

WAT was minced and digested at 37 °C (2 mg ml^–1^ collagenase II, 2 mg ml^–1^ dispase II and 2% BSA in PBS for sWAT; 1 mg ml^–1^ collagenase II, 1 mg ml^–1^ dispase II and 2% BSA in PBS for vWAT). Homogenates were filtered through 100-µm cell strainers (BD Falcon) and diluted 1:1 with PBS. Digests were centrifuged (300*g*, 5 min). Red blood cells were lysed in 1 ml of ACK lysis buffer (Thermo Fisher Scientific), followed by dilution with 10 ml of PBS. Cells were centrifuged (300*g*, 5 min) and suspended in 1 ml of PBS + 1% BSA). SVF cells were counted in a Neubauer counting chamber, and 3 × 10^5^ cells suspended on ice were stained with titrated antibody concentrations. Dead cells were excluded by DAPI staining (Carl Roth). Antibodies used are listed in the Reporting Summary. Aldefluor (Stem Cell Technologies, 01700) and Annexin-PI (BD Biosciences, 556547) kits were used according to the manufacturer’s instructions. ALDH activity was gated according to DEAB-treated controls. WAT ECs (CD31^+^CD45^–^DAPI^–^), macrophages (F4/80^+^CD11b^+^CD45^+^DAPI^–^) and stromal cells (CD140a^+^Sca1^+^CD31^–^CD45^–^DAPI^–^) were sorted into 1.5-ml tubes precoated with 2% BSA in PBS on a tube rotor for 4 h before sorting into 2% BSA in PBS. Cells rested on ice until centrifugation (5 min, 300*g*) and RNA isolation with a PicoPure RNA isolation kit (Thermo Fisher Scientific). Organoid cells were centrifuged following digestion and stained on ice using an anti-CD45 (Thermo Fisher Scientific, 12-0149-41).

### cDNA synthesis and qPCR

RNA quantity was measured using a Nanodrop 100 (Thermo Fisher Scientific). The High-Capacity cDNA Reverse Transcription Kit (Applied Biosystems) or the SuperScript IV VILO Master Mix (Life Technologies) were used for cDNA synthesis. cDNA was diluted 1:40 in water. mRNA expression was analyzed in doublets using SYBR Green master mix (Thermo Fisher Scientific) with either a QuantStudio 3 (Thermo Fisher Scientific) or StepOne Plus (Applied Biosystems). Gene expression was normalized relative to the housekeeping gene *OAZ1* for human ECs, *Cph*, *Rpl32* or *Rpl13* for mouse ECs and *Cph*, *Gapdh* or *Tuba* in whole WAT or adipocytes using the change in cycling threshold ($2^{-\Delta\Delta C_{t}}$) method. Primers used for RT–qPCR are listed in Supplementary Table 2.

### Immunohistochemistry

Paraffin-embedded samples were deparaffinized and rehydrated in xylene and step-wise reductions in alcohol concentrations. H&E staining was performed according to standard protocols. Antibodies and dilutions used are stated in the Reporting Summary. For Oil Red O staining, livers were preserved by cryofreezing. Stainings were performed on cryosections according to the manufacturer’s instructions (Sigma). Masson–Goldner trichrome staining was performed according to the manufacturer’s instructions (Carl Roth).

### Immunofluorescence

Spheroids were fixed in 4% paraformaldehyde, washed three times with PBS and blocked in 1% BSA in PBS and 0.1% Tween 20. Vessels were stained with primary anti-CD31 conjugated to Alexa Fluor 647 (1:500; Cell Signaling, 49940) in blocking buffer for 2 h at room temperature. Spheroids were washed twice with PBS, and LipidTOX (Life Technologies) and DAPI (Thermo Fisher Scientific) in blocking buffer were added for 15 min. Spheroids were washed twice with PBS and mounted in cavity slides (Marienfeld).

### Image acquisition and quantification

H&E and DAB stainings were acquired on an Axio Scan Slide Scanner Z.1 (Carl Zeiss). Whole-mount, organoid and immunofluorescence stainings (collagen IV and isolectin B4) were acquired on an LSM 700 confocal microscope (Carl Zeiss). Apoptosis/necrosis kit images were acquired on a Cell Observer (Carl Zeiss). Image analysis was performed using Fiji software^45^. The Adiposoft plugin was used for adipocyte area quantification. The Color Transformer plugin was used to quantify DAB, Oil Red O and Masson’s trichrome histological stainings. The JACoP plugin was used to measure collagen VI and isolectin B4 colocalization. The AnalyzeSkeleton plugin was used to measure reticular interstitium diameter.

### RNA-seq

Human AT-ECs (passage 0) from six human samples pooled per depot were treated with AdN1ICD or AdGFP adenoviruses in triplicates. RNA was isolated using an RNA Mini kit (Qiagen) and analyzed for quality control by Bioanalyzer (Agilent). Libraries were prepared by the DKFZ Genomics and Proteomics Core Facility. Libraries were pooled and sequenced in two lanes on an Illumina HiSeq 4000 (50-base pair single-end reads). Data were analyzed by the DKFZ Core Facility for Omics IT and Data Management to calculate RPKM values. Differentially expressed genes were identified using the DeSeq R package. GSEA and IPA were performed as described below.

### Microarray analysis

RNA from AT-ECs was isolated from KPC and control mice using an RNA Mini kit (Qiagen) and analyzed for quality control by Bioanalyzer (Agilent). RNA was hybridized to Mouse Affymetrix Clariom S Arrays, and data were subjected to normalization and group comparison by the DKFZ Genomics and Proteomics Core Facility. Differentially expressed genes were assessed by IPA analysis (*P* < 0.01; fold change > 1.35).

### GSEA

An ‘AT-EC Notch1 gene signature’ was created from the top 500 upregulated genes obtained from the RNA-seq data comparing vWAT AT-ECs overexpressing AdN1ICD to those expressing AdGFP. GSEA (Broad Institute) was used to determine enrichment of the ‘AT-EC Notch1 gene signature’, ‘hallmark apoptosis’, ‘hallmark adipogenesis’ and GO term ‘cellular response to RA’ in publicly available datasets consisting of microarray data from individuals with precachexia or cachexia (GEO GSE131835). Microarray data derived from whole vWAT from healthy control individuals and individuals with precachexia and cachexia and human AT-EC RNA-seq data were analyzed by GSEA with 1,000 phenotype permutations.

### IPA

IPA software was used to identify predicted upstream regulators in KPC AT-EC (microarray) and AdN1ICD AT-EC (RNA-seq) datasets. KPC AT-EC genes with a fold change of ≥1.35 and *P* value of <0.01 were used. The top 250 most significantly differentially expressed genes in AdNICD AT-ECs were used.

### Statistics and reproducibility

No statistical methods were used to predetermine sample sizes, but our sample sizes are similar to those reported in previous publications^11,18,25^. The experiments were not randomized. A minimum number of mice was bred in accordance with the 3R principle, and age-matched mice were selected from breedings. Researchers performing experiments with the C26 model were blind to the experimental hypothesis. Some western blots and RT–qPCR assays were performed by a researcher blind to the experimental hypothesis. In all other experiments, data collection and analyses were not performed blind to the conditions of the experiments. Animals were excluded for analyses in the case of failed tamoxifen-induced recombination (as confirmed by PCR) or if an animal needed to be removed from an experiment early to prevent disease burden. Due to WAT loss and lack of material, not all samples were included in all immunohistochemistry experiments, and stainings were performed based on availability of samples.

GraphPad Prism 9 was used to generate graphs and for statistical analyses. Groups were tested for normality. Statistical significance was calculated for two unmatched groups by unpaired, two-sided *t*-test with Welch’s correction or Mann–Whitney test. One- or two-way ANOVAs were used for more than two groups as specified, followed by Tukey’s multiple comparisons tests. Datasets are presented as mean ± s.e.m. *P* values under 0.05 were considered significant. Data distribution was assumed to be normal, but this was not formally tested.

### Schematic figures

Schematics were created using BioRender.com.

### Reporting summary

Further information on research design is available in the Nature Portfolio Reporting Summary linked to this article.

### Supplementary information

Reporting Summary

Supplementary Table Supplementary Tables 1 and 2.

### Source data

Source Data Fig. 1 Numerical data (graphs).

Source Data Fig. 2 Unprocessed western blots.

Source Data Fig. 2 Numerical data (graphs).

Source Data Fig. 3 Unprocessed western blots.

Source Data Fig. 3 Numerical data (graphs).

Source Data Fig. 4 Unprocessed western blots.

Source Data Fig. 4 Numerical data (graphs).

Source Data Fig. 5 Unprocessed western blots.

Source Data Fig. 5 Numerical data (graphs).

Source Data Fig. 6 Numerical data (graphs).

Source Data Fig. 7 Numerical data (graphs).

Source Data Fig. 8 Numerical data (graphs).

Source Data Extended Data Fig. 1 Numerical data (graphs).

Source Data Extended Data Fig. 2 Numerical data (graphs).

Source Data Extended Data Fig. 3 Numerical data (graphs).

Source Data Extended Data Fig. 4 Unprocessed western blots.

Source Data Extended Data Fig. 4 Numerical data (graphs).

Source Data Extended Data Fig. 5 Unprocessed western blots.

Source Data Extended Data Fig. 5 Numerical data (graphs).

Source Data Extended Data Fig. 6 Numerical data (graphs).

Source Data Extended Data Fig. 7 Numerical data (graphs).

Source Data Extended Data Fig. 8 Numerical data (graphs).

Source Data Extended Data Fig. 9 Numerical data (graphs).

## Data Availability

ChIP–seq, RNA-seq and microarray data supporting these findings have been deposited in the GEO under accession codes GSE195537 (ChIP–seq), GSE212926 (RNA-seq) and GSE212562 (microarray). Materials will be provided on reasonable request. Source data are provided with this paper. All other data are available in the manuscript or Supplementary Information.
