# Supplementary material for: Endothelial Notch1 signaling in white adipose tissue promotes cancer cachexia
Source: Nat Cancer. 2023 Sep 25;4(11):1544–60. doi: 10.1038/s43018-023-00622-y (PMC10663158; doi:10.1038/s43018-023-00622-y)
Supplement: Supplementary file 1 — Reporting Summary [file 43018_2023_622_MOESM1_ESM.pdf]

nature portfolio

Corresponding author(s): Juan Rodriguez-Vita; Andreas Fischer

Last updated by author(s): Jul 11, 2023

Reporting Summary

Nature Portfolio wishes to improve the reproducibility of the work that we publish. This form provides structure for consistency and transparency in reporting. For further information on Nature Portfolio policies, see our Editorial Policies and the Editorial Policy Checklist.

Statistics

For all statistical analyses, confirm that the following items are present in the figure legend, table legend, main text, or Methods section.

n/a	Confirmed	
☐	☒	The exact sample size (*n*) for each experimental group/condition, given as a discrete number and unit of measurement
☐	☒	A statement on whether measurements were taken from distinct samples or whether the same sample was measured repeatedly
☐	☒	The statistical test(s) used AND whether they are one- or two-sided<br>*Only common tests should be described solely by name; describe more complex techniques in the Methods section.*
☐	☒	A description of all covariates tested
☐	☒	A description of any assumptions or corrections, such as tests of normality and adjustment for multiple comparisons
☐	☒	A full description of the statistical parameters including central tendency (e.g. means) or other basic estimates (e.g. regression coefficient) AND variation (e.g. standard deviation) or associated estimates of uncertainty (e.g. confidence intervals)
☐	☒	For null hypothesis testing, the test statistic (e.g. *F*, *t*, *r*) with confidence intervals, effect sizes, degrees of freedom and *P* value noted<br>*Give P values as exact values whenever suitable.*
☒	☐	For Bayesian analysis, information on the choice of priors and Markov chain Monte Carlo settings
☒	☐	For hierarchical and complex designs, identification of the appropriate level for tests and full reporting of outcomes
☒	☐	Estimates of effect sizes (e.g. Cohen's *d*, Pearson's *r*), indicating how they were calculated

Our web collection on statistics for biologists contains articles on many of the points above.

Software and code

Policy information about availability of computer code

Data collection	Microscopy images were acquired using ZEN blue Cell Observer (Zeiss) or Zen black by LSM700, LSM710, Axio Scan Slide Scanner Z.1 or Cell Observer (Carl Zeiss). Flow cytometry data was collected using FACS Diva software (BD Biosciences). qRT-PCR data was collected using QuantStudio 3 (Thermo Fisher), StepOne Plus (Agilent) or Light Cycler480 II (Roche) softwares. Western blot images were collected using ImageLab software (Biorad).
Data analysis	Raw data from technical replicates were analysed using Excel (Microsoft) prior to statistical analysis of biological replicates using Graphpad Prism v9 software. Experiments involving technical replicates were averaged to one biological replicate unless otherwise indicated (adipocyte quantification). Microscopy images were processed and analysed using ZEN blue (Zeiss), ZEN black (Zeiss) and Fiji software using Adiposoft, Color Transformer, JACoP and AnalyzeSkeleton plugins as indicated under Methods. Gene set enrichment analysis (GSEA) was performed using GSEA software (v4.0.3, Broad Institute). Heatmaps were generated from GSEA software or from normalized data using R Studio (v1.2.5033) with the ComplexHeatmap package. RNAseq data was analysed using the DESeq package to obtain differentially expressed genes. Ingenuity pathway analysis (Qiagen) was used to identify top predicted upstream regulators based on differentially expressed genes. FlowJo v10 was used for flow cytometry analysis.<br><br>ChipSeq: Raw FASTQ files were quality and adaptor trimmed using trimGalore v.0.6.5 . Trimmed files were aligned against the human reference genome (hg19) using Hisat2 v.2.2.152 and stored as binary alignment maps (BAM). Quality of the alignment was inspected and validated within R v.4.0.2 using systemPipeR's alignStats function. PCR duplicates were removes using Picard tools. Coverage tracks based on the processed BAM files were generated using Deeptools bamCoverage and stored as BigWig files (RPKM normalized). Binding profiles were visualized within R using the R/BioConductor54 package GVIZ.

For manuscripts utilizing custom algorithms or software that are central to the research but not yet described in published literature, software must be made available to editors and reviewers. We strongly encourage code deposition in a community repository (e.g. GitHub). See the Nature Portfolio guidelines for submitting code & software for further information.

Data

Policy information about availability of data

All manuscripts must include a data availability statement. This statement should provide the following information, where applicable:

- Accession codes, unique identifiers, or web links for publicly available datasets
- A description of any restrictions on data availability
- For clinical datasets or third party data, please ensure that the statement adheres to our policy

All data are available in the manuscript or supplementary figures. The GEO accession number is GSE195537 for the ChIP-Seq, GSE212926 for RNA-Seq and GSE212562 for microarray data. Materials will be provided on reasonable request.

Human research participants

Policy information about studies involving human research participants and Sex and Gender in Research.

Reporting on sex and gender	Using publicly available human datasets, we performed some analyses (GSEA) in groups separated based on the patient's sex. (GEO: GSE131835).<br>Human adipose tissue biopsies were obtained from both sexes to isolated AT-ECs for cell culture experiments. Researchers were blind to the sex of the patient and samples were pooled from both sexes.
Population characteristics	Data was analysed was from a publicly available dataset in which age and sex are listed by authors . Samples were not selected based on age given that less than 10 per group were available.(GEO: GSE131835).<br>AT-ECs were isolated from adipose tissue depots of human visceral abdominal and subcutaneous abdominal adipose tissue biopsies and collected from patients undergoing bariatric surgery at the University Hospital Heidelberg. Patient samples were pooled depending on the number of patient samples collected (up to six). For RNA-seq experiments, AT-ECs were isolated from six patients and pooled to mask the identity of individual samples, and subsequently, treated with AdGFP of AdN1ICD adenoviruses to overexpress GFP or N1ICD.
Recruitment	ECs were isolated from adipose tissue depots of human visceral abdominal and subcutaneous abdominal adipose tissue biopsies and collected from patients undergoing bariatric surgery at the Department of Surgery, University Hospital Heidelberg.
Ethics oversight	Collection was approved by the Institutional Review Board of the Medical Faculty of the University of Heidelberg in accordance with the Declaration of Helsinki. All patients gave informed preoperative consent prior to sample collection.

Note that full information on the approval of the study protocol must also be provided in the manuscript.

Field-specific reporting

Please select the one below that is the best fit for your research. If you are not sure, read the appropriate sections before making your selection.

☒ Life sciences ☐ Behavioural & social sciences ☐ Ecological, evolutionary & environmental sciences

For a reference copy of the document with all sections, see nature.com/documents/nr-reporting-summary-flat.pdf

Life sciences study design

All studies must disclose on these points even when the disclosure is negative.

Sample size	No stastical methods were used to determine sample size. Sample sizes were determined based on experience and variability of previously published experiments (Wieland et al., Cancer Cell, 2017, Jabs et al. Circ, 2018, Hasan et al. Embo Mol Med 2019). All sample sizes are indicated in figure legends.
Data exclusions	Data were excluded if technical problems were detected (failed genetic recombination confirmed by PCR or severe deviations in technical replicates). Some NICDiOE-EC numbers (week 7) were excluded from some immunohistochemistry staining experiments due to lack of material caused by fat loss in mice. Mice were excluded from KPC experiments if they had to be sacrificed early for ethical reasons. All repetitions of experiments were consistent.
Replication	All experiments were performed and calculated based on biological replicates. All in vitro experiments were repeated three times unless otherwise indicated (phenotype timeline- week 2, Extended Data Fig. 2). Human-derived samples were obtained from different patients per biological replicate, including AT-ECs and organoids. Conclusions on the link to cachexia were made based on 2 different experimental models at 2 separate institutions (Heidelberg and Munich). All attempts at replication were consistent. In some NICDiOE-EC experiments, samples from two experiments were pooled.
Randomization	NICDiOE-EC and RbpjiEC mice were littermates (cre- or cre+) and therefore, no randomization was required. No statistical methods were used to pre-determine sample sizes but our sample sizes are similar to those reported in previous publications. A minimum number of transgenic

mice were bred in accordance with the 3R principle and mice were selected for experiments based on age matching. Wildtype mice used for cachexia experiments were assigned randomly based on age matching. Human cells derived from the same patient(s) were used for both control and experimental conditions in all in vitro experiments.

Blinding

Researchers performing C26 cachectic experiments and analyses were blind to the experimental hypotheses. Investigators were not blind to genotype during mouse experiments due to a clear phenotype. Some Western blots and qRT-PCRs were performed by a researcher blind to the experimental hypotheses. Researchers were not blind in other experiments.

Reporting for specific materials, systems and methods

We require information from authors about some types of materials, experimental systems and methods used in many studies. Here, indicate whether each material, system or method listed is relevant to your study. If you are not sure if a list item applies to your research, read the appropriate section before selecting a response.

Materials & experimental systems

n/a	Involved in the study
☐	☒ Antibodies
☐	☒ Eukaryotic cell lines
☒	☐ Palaeontology and archaeology
☐	☒ Animals and other organisms
☒	☐ Clinical data
☒	☐ Dual use research of concern

Methods

n/a	Involved in the study
☐	☒ ChIP-seq
☐	☒ Flow cytometry
☒	☐ MRI-based neuroimaging

Antibodies

Antibodies used

All antibodies are from commercial sources. Detailed information on their use is provided within the Methods section.
Western blot primary antibodies: ALDH1A2 (Cell Signaling, 83805S, 1:1000), Arginase-1 (Cell Signaling, 93668, 1:1000), Cleaved Caspase-3 Asp175 (Cell Signaling, 9664, 1:1000), IL-33 (Abcam, ab54385, 1:1000), TAGLN (Abcam, ab137453, 1:1000), VCAM-1 (Abcam, ab134047, 1:1000), VCP (Abcam, ab11433, 1:5000), β-actin (Sigma, A5441, 1:2000), UCP1 (Cell Signaling, 14670, 1:1000)
Western blot secondary antibodies: Polyclonal Goat Anti-Rabbit HRP (Dako, P0448, 1:2500), Polyclonal Rabbit Anti Mouse HRP rabbit (Dako, P0260, 1:2500)
AT-EC isolation (KPC and week 7 AT-ECs): Rat-anti mouse CD45 antibody (BD Biosciences, 553078, 1:200), rat anti-mouse CD31 antibody (BD Biosciences, 550274, 1:200), Dynabeads-human CD31 (Thermo Fisher, 11155D, 1:150), Dynabeads Sheep Anti-Rat IgG (Thermo Fisher, 11035, 1:150)
Flow cytometry and FACS: Antibodies from BD Biosciences: CD31 (561814), CD45 (552848, 550994, 552848, 612975), CD11b (5528520, 741934), F4/80 (565410), SiglecF (740280), Ly6C (560592), Ly6G (560600). Antibodies from Thermo Fisher Scientific: CD34 (48-0341-82). Antibodies from Biolegend: CD140a (135905), Sca1 (108123), F4/80 (123129). Aldefluor (Stem Cell Technologies, 01700) and Annexin-PI (BD Biosciences, 556547) kits were used according to manufacturer's instructions.
Flow cytometry (human): anti-human CD45 (BD Biosciences, 561863, 1:100)
Immunohistochemistry: CD11b (abcam, ab133357, 1:200), TAGLN (Abcam, ab127453, 1:100), collagen IV antibody (Biorad, 2150-1470, 1:200), isolectin B4 Alexa 647 (Thermo Fisher Scientific, I32450, 1:200), Goat anti-rabbit Alexa 546 (Thermo Fisher Scientific, A21245, 1:200), Goat anti-rabbit HRP (Dako, P0448, 1:200), Rabbit anti-mouse HRP (Dako, P0260, 1:200)
Immunofluorescence: CD31 (Cell Signaling, 49940, 1:500), UCP1 (Abcam, ab23841, 1:100), IGFBP3 (LS-Bio, LS-B12492)
Chip-Seq: H3K27ac antibody (Diagenode, pAb-174-050, 2.5 ug / 100 ug (antibody / chromatin ratio)), His2Av antibody (Active Motif, 61686, 1 ug / 100 ug (antibody / chromatin ratio))

Validation

All antibodies were validated by the manufacturers (Cell Signaling, Abcam, Sigma, Dako, BD Biosciences,Thermo Fisher, Biolegend, BioRad, LS-Bio, Diagenode, Active Motif) for antigen specificity and species reactivity as shown in the data sheets and attached references for each catalogue number. All stainings showed specific labelling similiar to validated results from the manufacturers. Antibodies used for Western blot were confirmed to have the correct molecular weights.

Eukaryotic cell lines

Policy information about cell lines and Sex and Gender in Research

Cell line source(s)

HEK293A cells were obtained as part of the ViraPower Adenoviral Expression System (Thermo Fisher). Primary endothelial cells were isolated from human adipose tissue biopsies for in vitro experiments. Human and mouse stromal vascular fractions were isolated from vWAT and/or sWAT. Human AT-ECs were used up to passage 5. Human AT-ECs treated with recombinant TNF-alpha were purchased from Innoprot (Spain). KPC cell line was provided by Prof. Stephen Konieczny, at Purdue University. Drosophila melanogaster Schneider cells were a gift of Rainer Renkawitz and Marek Bartkuhn (University of Giessen).

Authentication

HEK293A cells (Thermo Fisher) and KPC cells were not authenticated. Human endothelial cell identity was confirmed through immunofluorescence based stainings of classical endothelial cell markers (VE-cadherin, CD31). Drosophila melanogaster Schneider cells were validated via sequencing.

Mycoplasma contamination

HEK293A and KPC cells were routinely tested for myoplasma contamination by PCR for mycoplasma contamination (primers:

Mycoplasma contamination	gggagcaaacaggattagataccct, tcggaccatcatctgtcactctgttaacctc). Primary cells were not tested.
Commonly misidentified lines (See ICLAC register)	No misidentified lines were used in this study.

Animals and other research organisms

Policy information about studies involving animals; ARRIVE guidelines recommended for reporting animal research, and Sex and Gender in Research

Laboratory animals

Animals were housed under specific pathogen-free barrier conditions and fed ad libitum a standard mouse chow (3437, Granovit AG). Animals were housed at 22 ± 2 °C with 60 % humidity and a 12-hour light /dark rhythm.
NICDiOE-EC mice: Flox-Notch1-ICD (Jackson Laboratory, USA) crossed with Cdh5(PAC)CreERT2 mice (C57BL/6J background). NICDiOE-EC mice and controls (cre-) were given tamoxifen at 8-10 weeks of age (both sexes) and were used for experiments 2-7 weeks later.
RbpjiΔEC mice: tamoxifen-inducible, EC-specific Rbpjκ deletion was induced in Rbpjlox/lox mice (Jackson Laboratory, USA) crossed with Cdh5(PAC)CreERT2 mice16,22 (C57BL/6N background). Tamoxifen dissolved in peanut oil was administered orally (2 mg for three consecutive days) at 9-13 weeks of age. Control mice which did not express CreERT2 were also treated with tamoxifen.
RbpjiΔEC mice were injected with PBS or KPC cells at three weeks following oral administration of tamoxifen.

KPC model: Mice were injected with cells from the pancreatic ductal adenocarcinoma cell line derived from KPC mice (KrasG12D; Trp53R172H; Elas-CreER)50. 106 KPC cells in 100 μL PBS were injected intraperitoneally into 9-14 week old C57BL/6J or RbpjiΔEC mice and compared to PBS-injected, age-matched littermate controls. Tumour growth and animal well-being were closely inspected daily according to score sheets monitoring variables such as body weight, behaviour and tumour growth (by palpations). To analyse gene expression during pre-cachexia (<10% change in body mass), mice were analysed at 11 days post-tumour injection.

C26 BALB/c male mice were injected with tumor cells at 10-14 weeks of age. Mice were sacrificed 16-21 days after tumor cell injection.
8-10 week old male C57BL/6J mice (Janvier) were used for in vitro experiments involving SVF cells.

Wild animals	The study did not involve wild animals.
Reporting on sex	Sex was considered in all aspects of the study. NICDiOE-EC experiments were performed in both sexes to analyse phenotype. A phenotype was only seen in male mice. Based on this, only male mice were used for the C26 and KPC models as the purpose was to analyse downstream components of the Notch pathway which contribute to WAT remodelling.
Field-collected samples	The study did not involve field-collected animals.
Ethics oversight	All animal procedures were performed in accordance with institutional and national regulations and approved by local committees for animal experimentation (RP Karlsruhe, DKFZ, Heidelberg University and RP Upper Bavaria).

Note that full information on the approval of the study protocol must also be provided in the manuscript.

ChIP-seq

Data deposition

☒ Confirm that both raw and final processed data have been deposited in a public database such as GEO.

☒ Confirm that you have deposited or provided access to graph files (e.g. BED files) for the called peaks.

Data access links
May remain private before publication.

https://www.ncbi.nlm.nih.gov/geo/query/acc.cgi?acc=GSE195537

Files in database submission

H3K27ac_GFP_Rep1_1.fastq.gz
H3K27ac_GFP_Rep1_2.fastq.gz
H3K27ac_GFP_Rep2_1.fastq.gz
H3K27ac_GFP_Rep2_2.fastq.gz
H3K27ac_N1ICD_Rep1_1.fastq.gz
H3K27ac_N1ICD_Rep1_2.fastq.gz
H3K27ac_N1ICD_Rep2_1.fastq.gz
H3K27ac_N1ICD_Rep2_2.fastq.gz
Input_GFP_1.fastq.gz
Input_GFP_2.fastq.gz
Input_N1ICD_1.fastq.gz
Input_N1ICD_2.fastq.gz

H3K27ac_GFP_1.bw
H3K27ac_GFP_2.bw
H3K27ac_N1ICD_1.bw
H3K27ac_N1ICD_2.bw
Input_GFP.bw

	Input_N1ICD.bw
Genome browser session (e.g. UCSC)	https://genome.ucsc.edu/s/tobiasfrie/NICD_Cach

Methodology

Replicates	Two biological replicates from adipose tissue endothelial cells infected with with adenoviruses expressing the GFP protein or N1ICD protein.
Sequencing depth	Paired End reads:<br>FileName Nreads2x Nalign Perc_Aligned<br>GFP_1 78436180 73942231 94.2705662106441<br>N1ICD_1 78012740 72706547 93.1982994059688<br>GFP_2 69037542 64956928 94.0892826109018<br>N1ICD_2 59630912 55387653 92.8841286210749<br>Input_GFP 64599502 58858071 91.1122673979747<br>Input_N1ICD 67400518 61241864 90.8626013823811
Antibodies	H3K27ac: Diagenode pAb-174-050
Peak calling parameters	Read mapping was performed using HISAT2 v.2.2.1 with parameter "--no-spliced-alignment" against hg19. Peak calling was performed using MACS2 v.2.2.7.1 with input (GFP or NICD), q-value < 0.01 and genome size 2.7e9. Peaks were filtered for blacklisted regions.
Data quality	Reads were quality and adapter trimmed using TrimGalore v.0.6.4. with standard parameters. FASTQ files were inspected using fastqc. Peaks were optically validated using the genome browser. Peaks had to be conserved in both replicates to be accepted.
Software	fastqc (https://www.bioinformatics.babraham.ac.uk/projects/fastqc/)<br>trimGalore (https://www.bioinformatics.babraham.ac.uk/projects/trim_galore/)<br>HISAT2 (http://daehwankimlab.github.io/hisat2/)<br>IGV (https://software.broadinstitute.org/software/igv/)<br>deepTools (https://deeptools.readthedocs.io/en/develop/)<br>MACS2 (https://github.com/hbctraining/Intro-to-ChIPseq)<br>BioConducter GenomicRanges (https://bioconductor.org/packages/release/bioc/html/GenomicRanges.html)

Flow Cytometry

Plots

Confirm that:

☒ The axis labels state the marker and fluorochrome used (e.g. CD4-FITC).

☒ The axis scales are clearly visible. Include numbers along axes only for bottom left plot of group (a 'group' is an analysis of identical markers).

☒ All plots are contour plots with outliers or pseudocolor plots.

☒ A numerical value for number of cells or percentage (with statistics) is provided.

Methodology

Sample preparation	WAT was minced and digested at 37°C (2 mg/mL collagenase II, 2 mg/ml dispase II, 2% BSA in PBS for sWAT; 1 mg/mL collagenase II, 1 mg/mL dispase II, 2% BSA in PBS for vWAT). Homogenates were filtered through 100 µM cell strainers (BD Falcon) and diluted 1:1 with PBS. Digests were centrifuged (300xg, 5 min.). RBCs were lysed in 1mL ACK lysis buffer (Thermo Fisher Scientific) followed by dilution with 10 mL PBS. Cells were centrifuged (300xg, 5 min.) and suspended (1 mL PBS + 1% BSA). SVF cells were counted in a Neubauer counting chamber and 3x105 suspended on ice were stained with titrated antibody concentrations. Dead cells were excluded by DAPI staining (Carl Roth). Antibodies from BD Biosciences: CD31 (561814), CD45 (552848, 550994, 552848, 612975), CD11b (5528520, 741934), F4/80 (565410), SiglecF (740280), Ly6C (560592), Ly6G (560600). Antibodies from Thermo Fisher Scientific: CD34 (48-0341-82). Antibodies from Biolegend: CD140a (135905), Sca1 (108123), F4/80 (123129). Aldefluor (Stem Cell Technologies, 01700) and Annexin-PI (BD Biosciences, 556547) kits were used according to manufacturer's instructions. ALDH activity was gated according to DEAB-treated controls. WAT ECs (CD31+CD45-DAPI-), macrophages (F4/80+CD11b+CD45+DAPI-) and stromal cells (CD140a+Sca1+CD31-CD45-DAPI-) were sorted into 1.5 mL tubes pre-coated with 2% BSA in PBS at a tube rotor for 4 hours prior to sorting into 2% BSA in PBS. Cells rested on ice until centrifugation (5 minutes, 300xg) and RNA isolation with the PicoPure RNA isolation kit (Thermo Fisher Scientific). Organoid cells were centrifuged following digestion and stained on ice using CD45 (Thermo Fisher Scientific, 12-0149-41).
Instrument	For flow cytometry analysis: FACS Canto II (BD Biosciences), LSR Fortessa (BD Biosciences)<br>For cell sorting: FACS Aria (BD Biosciences)

Software

Analyses were performed with FlowJo (v9) and Graphpad Prism (v9).

Cell population abundance

Debris and single cells were excluded by FSC and SSC.
AT-EC sorting: DAPI- cells (to exclude dead cells) -> CD31 vs CD45 (to select for CD31+CD45- endothelial cells)
Macrophage sorting:DAPI- cells (to exclude dead cells) -> CD31 vs CD45 (to select for CD31-CD45+ immune cells) ->CD11bvsF4/80 (to select for CD11b+CD45+F4/80hi macrophages)
Stromal cell sorting:DAPI- cells (to exclude dead cells) -> CD31 vs CD45 (to select for CD31-CD45- cells) ->CD140a vs Sca1 (to select for CD140a+Sca1+ stromal cells)
AT-EC Aldefluor analysis: CD31 vs CD45 (to select for CD31+CD45- endothelial cells) -> FSC-H vs ALDH (to determine ALDHhi ECs, gating performed based on DEAB inhibiting control)
Macrophages (with or without Aldefluor analysis): FSC-H vs CD45 (to select for CD45+ immune cells) -> CD11b vs F4/80 (to select for CD11b+F4/80hi macrophages) -> FSC-H vs ALDH (to determine ALDHhi macrophages, gating performed based on DEAB inhibiting control)
Eosinophils (with or without Aldefluor analysis): FSC-H vs CD45 (to select for CD45+ immune cells) -> CD11b vs SiglecF (to select for CD11b+SiglecF eosinophils) -> FSC-H vs ALDH (to determine ALDHhi eosinophils gating performed based on DEAB inhibiting control)
Neutrophils (with or without Aldefluor analysis): FSC-H vs CD45 (to select for CD45+ immune cells) -> CD11b vs Ly6G (to select for CD11b+Ly6G+ neutrophils -> FSC-H vs ALDH (to determine ALDHhi neutrophils gating performed based on DEAB inhibiting control)
Monocytes (with or without Aldefluor analysis): FSC-H vs CD45 (to select for CD45+ immune cells) -> CD11b vs Ly6G (to select for CD11b+Ly6G- cells) -> CD11b vs Ly6C (to select for CD11b+Ly6C- monocytes -> FSC-H vs ALDH (to determine ALDHhi monocytes gating performed based on DEAB inhibiting control)
Progenitor apoptosis: CD31 vs CD45 (to select for CD31-CD45- non-immune or endothelial cells) -> FSC-H vs CD34 (to select for remaining CD34+ progenitors) -> AnnexinV vs PI (to determine early apoptosis (Annexin V+PI-) late apoptosis (Annexin V +PI+), necrosis (Annexin V-PI+))
Stromal cell apoptosis:DAPI- cells (to exclude dead cells) -> CD31 vs CD45 (to select for CD31-CD45- cells) ->CD140a vs Sca1 (to select for CD140a+Sca1+ stromal cells) -> AnnexinV vs PI (to determine early apoptosis (Annexin V+PI-) late apoptosis (Annexin V+PI+), necrosis (Annexin V-PI+))
Organoid Aldefluor macrophage analysis: FSC-H vs CD45 (to select for CD45+ immune cells) -> FSC-H vs ALDH (to determine ALDHhi macrophages, gating performed based on DEAB inhibiting control)
Organoid Aldefluor immune cell analysis: FSC-H vs CD45 (to select for CD45+ immune cells) -> FSC-H vs ALDH (to determine ALDHhi immune cells, gating performed based on DEAB inhibiting control)

Gating strategy

Debris and single cells were excluded by FSC and SSC.
AT-EC sorting: DAPI- cells (to exclude dead cells) -> CD31 vs CD45 (to select for CD31+CD45- endothelial cells)
Macrophage sorting:DAPI- cells (to exclude dead cells) -> CD31 vs CD45 (to select for CD31-CD45+ immune cells) ->CD11bvsF4/80 (to select for CD11b+CD45+F4/80hi macrophages)
Stromal cell sorting:DAPI- cells (to exclude dead cells) -> CD31 vs CD45 (to select for CD31-CD45- cells) ->CD140a vs Sca1 (to select for CD140a+Sca1+ stromal cells)
AT-EC Aldefluor analysis: CD31 vs CD45 (to select for CD31+CD45- endothelial cells) -> FSC-H vs ALDH (to determine ALDHhi ECs, gating performed based on DEAB inhibiting control)
Macrophages (with or without Aldefluor analysis): FSC-H vs CD45 (to select for CD45+ immune cells) -> CD11b vs F4/80 (to select for CD11b+F4/80hi macrophages) -> FSC-H vs ALDH (to determine ALDHhi macrophages, gating performed based on DEAB inhibiting control)
Eosinophils (with or without Aldefluor analysis): FSC-H vs CD45 (to select for CD45+ immune cells) -> CD11b vs SiglecF (to select for CD11b+SiglecF eosinophils) -> FSC-H vs ALDH (to determine ALDHhi eosinophils gating performed based on DEAB inhibiting control)
Neutrophils (with or without Aldefluor analysis): FSC-H vs CD45 (to select for CD45+ immune cells) -> CD11b vs Ly6G (to select for CD11b+Ly6G+ neutrophils -> FSC-H vs ALDH (to determine ALDHhi neutrophils gating performed based on DEAB inhibiting control)
Monocytes (with or without Aldefluor analysis): FSC-H vs CD45 (to select for CD45+ immune cells) -> CD11b vs Ly6G (to select for CD11b+Ly6G- cells) -> CD11b vs Ly6C (to select for CD11b+Ly6C- monocytes -> FSC-H vs ALDH (to determine ALDHhi monocytes gating performed based on DEAB inhibiting control)
Progenitor apoptosis: CD31 vs CD45 (to select for CD31-CD45- non-immune or endothelial cells) -> FSC-H vs CD34 (to select for remaining CD34+ progenitors) -> AnnexinV vs PI (to determine early apoptosis (Annexin V+PI-) late apoptosis (Annexin V +PI+), necrosis (Annexin V-PI+))
Stromal cell apoptosis:DAPI- cells (to exclude dead cells) -> CD31 vs CD45 (to select for CD31-CD45- cells) ->CD140a vs Sca1 (to select for CD140a+Sca1+ stromal cells) -> AnnexinV vs PI (to determine early apoptosis (Annexin V+PI-) late apoptosis (Annexin V+PI+), necrosis (Annexin V-PI+))
Organoid Aldefluor macrophage analysis: FSC-H vs CD45 (to select for CD45+ immune cells) -> FSC-H vs ALDH (to determine ALDHhi macrophages, gating performed based on DEAB inhibiting control)
Organoid Aldefluor immune cell analysis: FSC-H vs CD45 (to select for CD45+ immune cells) -> FSC-H vs ALDH (to determine ALDHhi immune cells, gating performed based on DEAB inhibiting control)

☒ Tick this box to confirm that a figure exemplifying the gating strategy is provided in the Supplementary Information.
