# Supplementary figures and images for: Endothelial Notch1 signaling in white adipose tissue promotes cancer cachexia
Source: Nat Cancer. 2023 Sep 25;4(11):1544–60. doi: 10.1038/s43018-023-00622-y (PMC10663158; doi:10.1038/s43018-023-00622-y)

Figure 2i

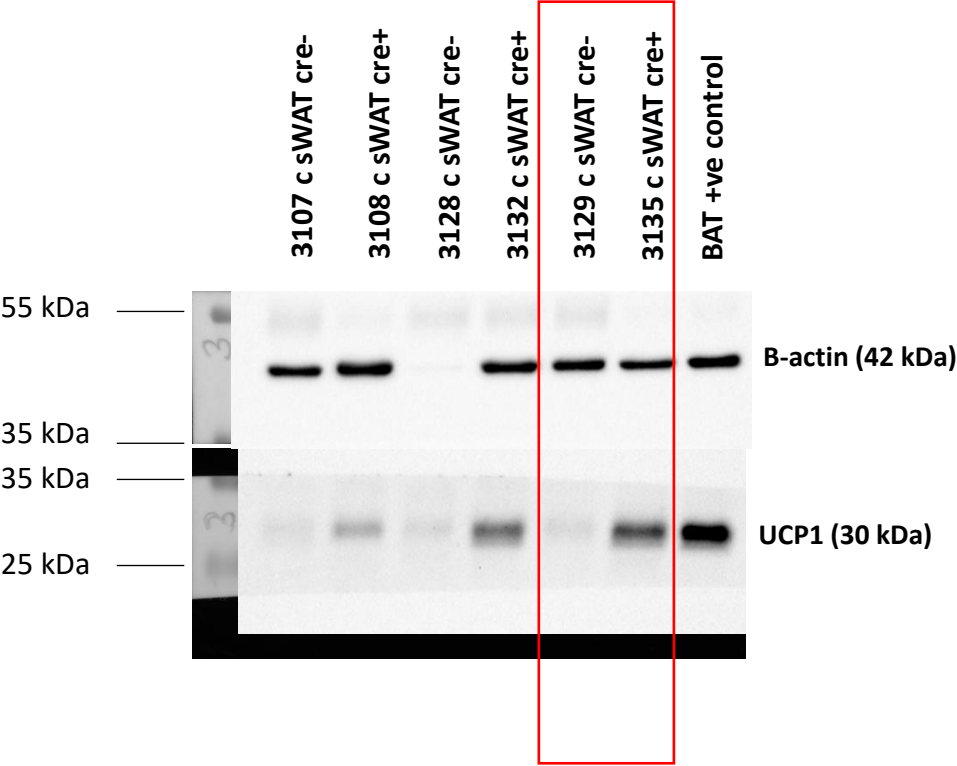

Figure 2o

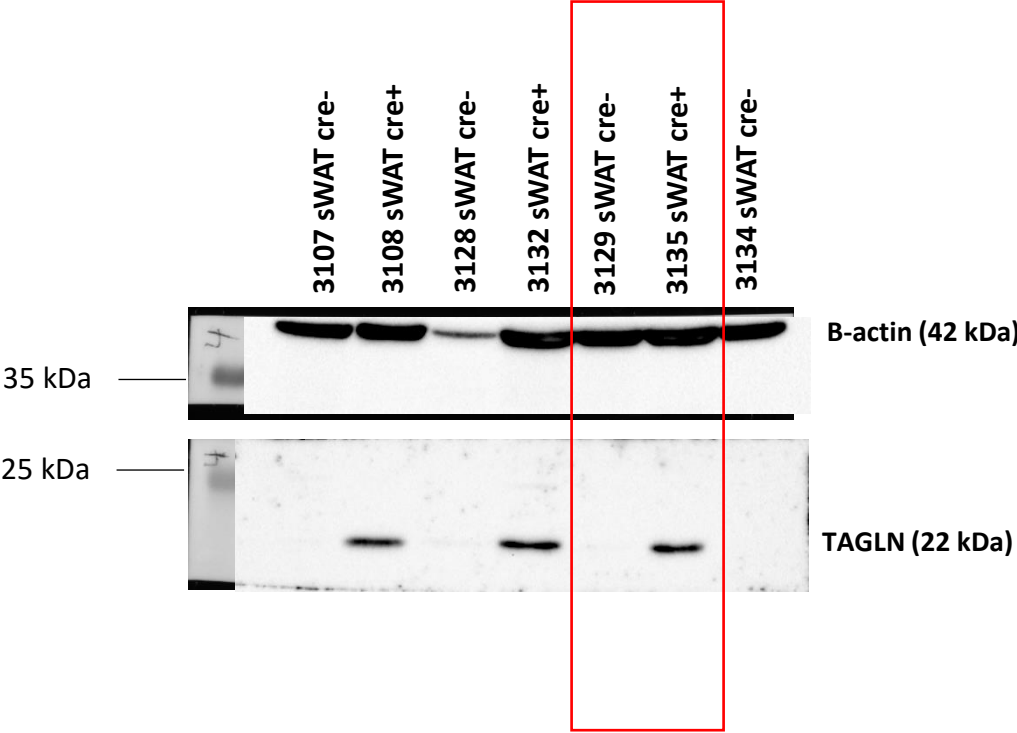

Supplement: Supplementary file 4 — Unprocessed western blots. [file 43018_2023_622_MOESM4_ESM.pdf]

Figure 3g

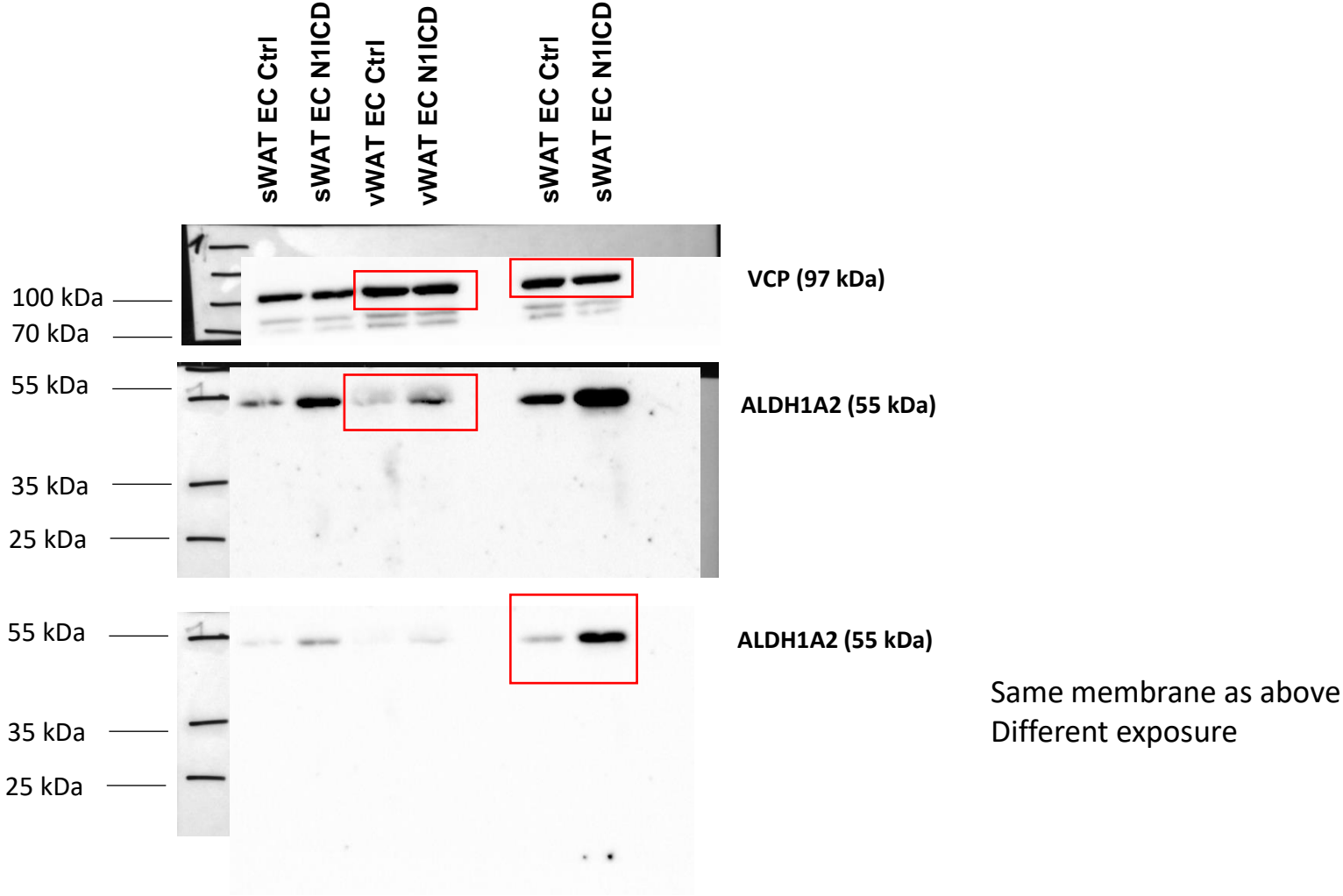

Supplement: Supplementary file 6 — Unprocessed western blots. [file 43018_2023_622_MOESM6_ESM.pdf]

Figure 4c

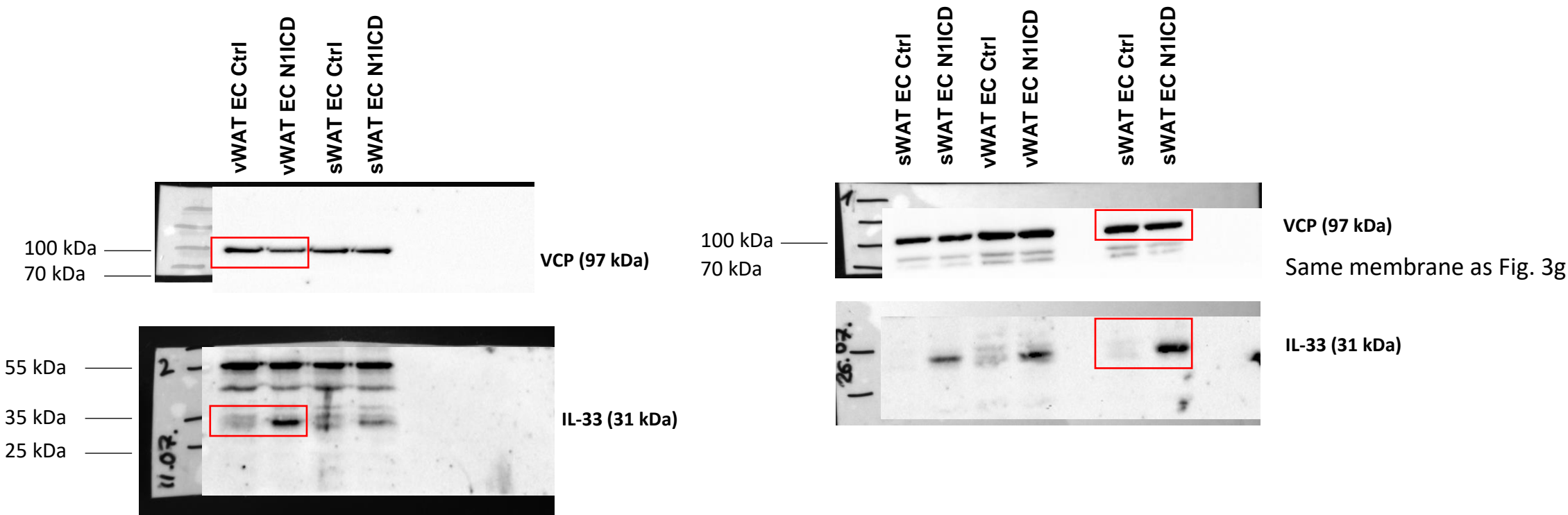

Supplement: Supplementary file 8 — Unprocessed western blots. [file 43018_2023_622_MOESM8_ESM.pdf]

Fig. 5b

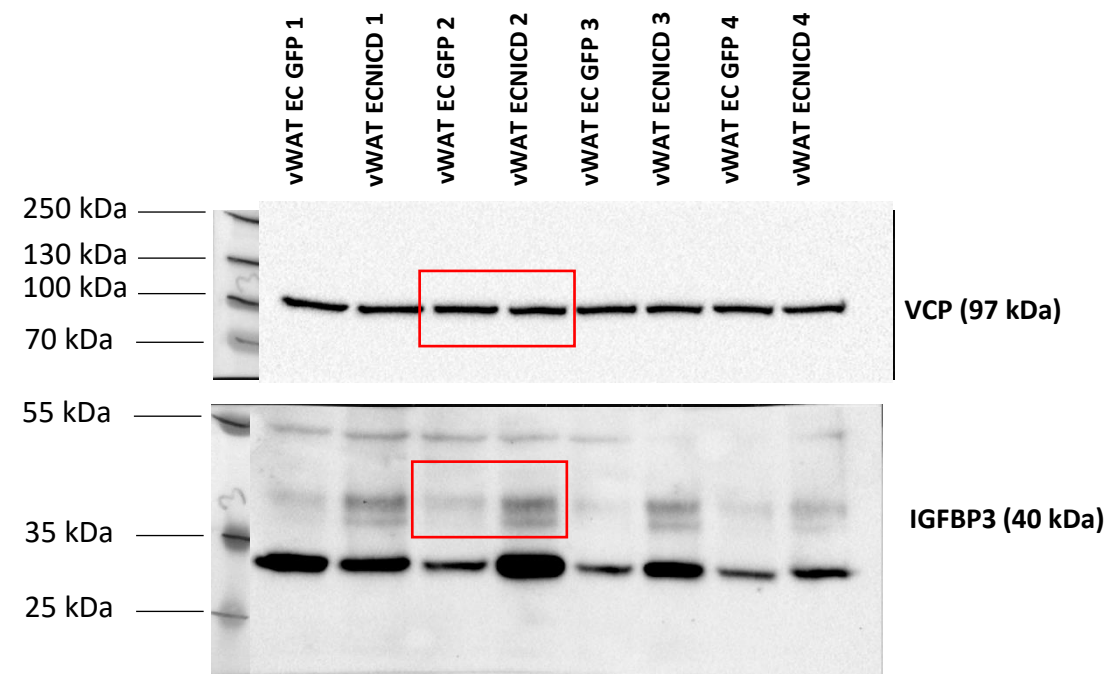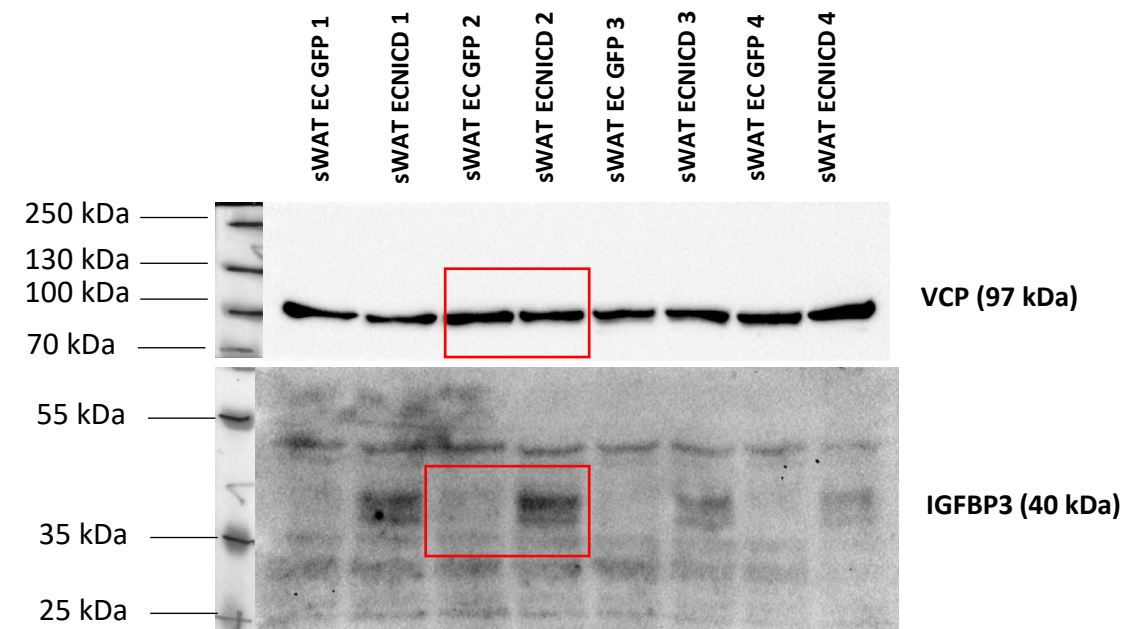

Fig. 5f

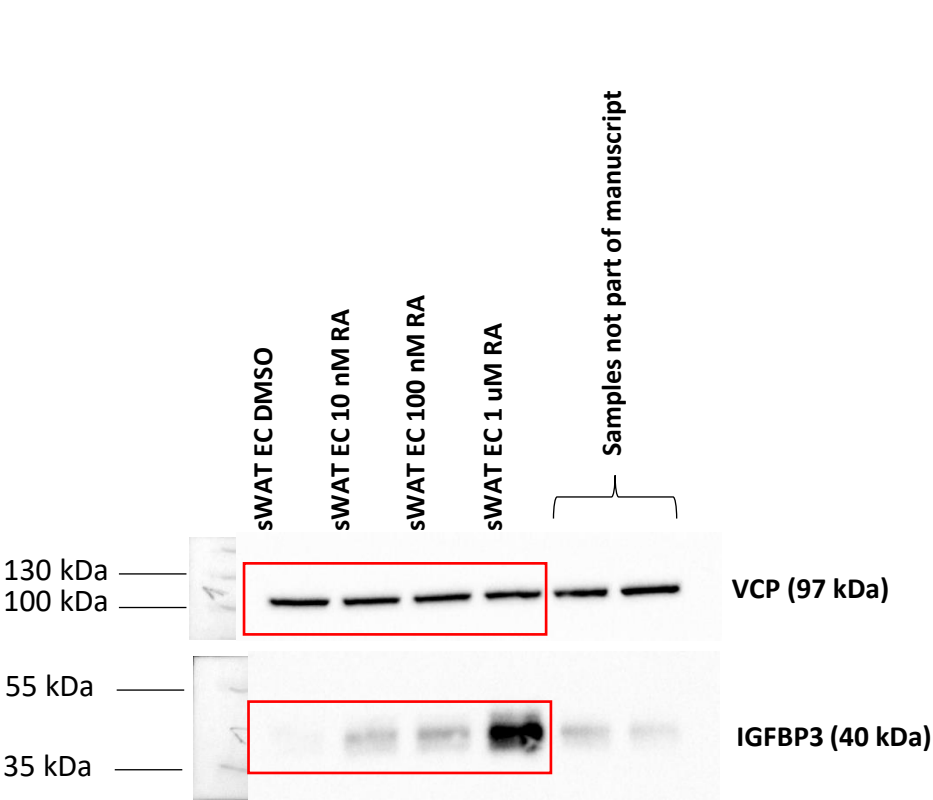

Fig. 5m

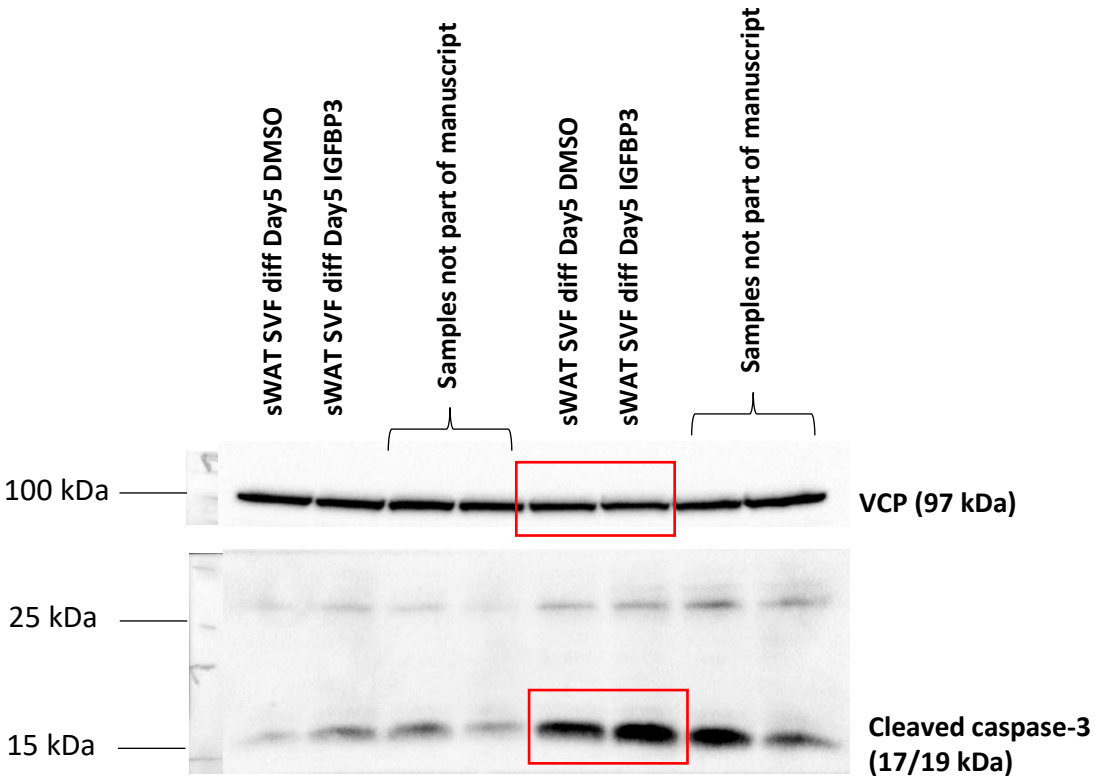

Supplement: Supplementary file 10 — Unprocessed western blots. [file 43018_2023_622_MOESM10_ESM.pdf]

Extended Data Fig. 4j

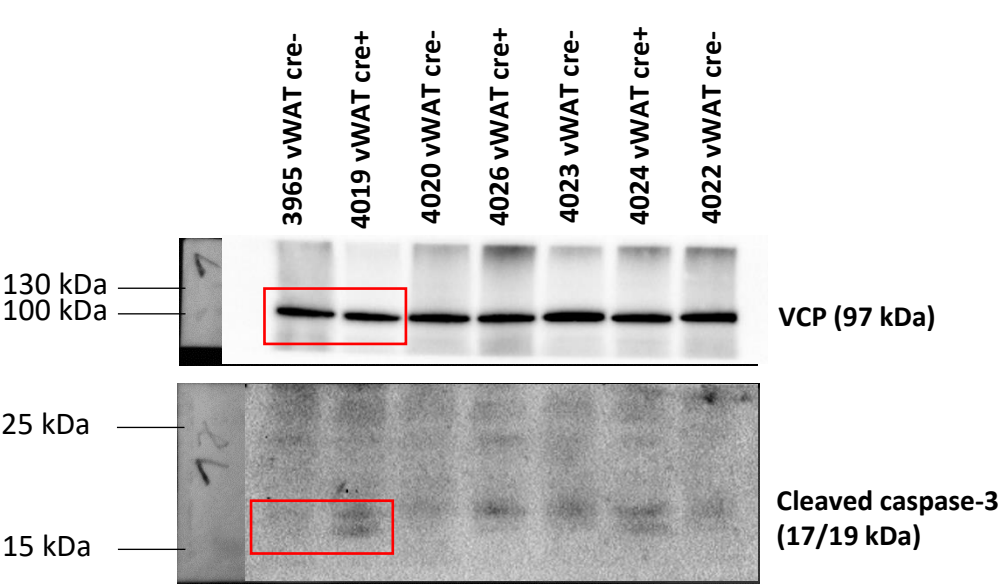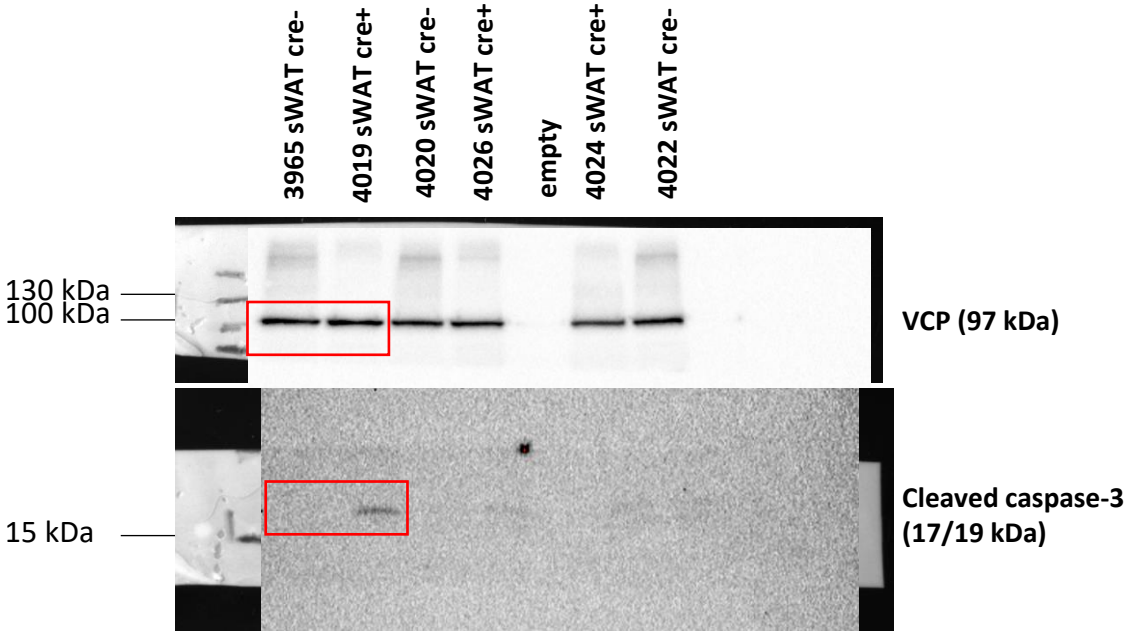

Supplement: Supplementary file 18 — Unprocessed western blots. [file 43018_2023_622_MOESM18_ESM.pdf]

Extended Data Fig. 5k

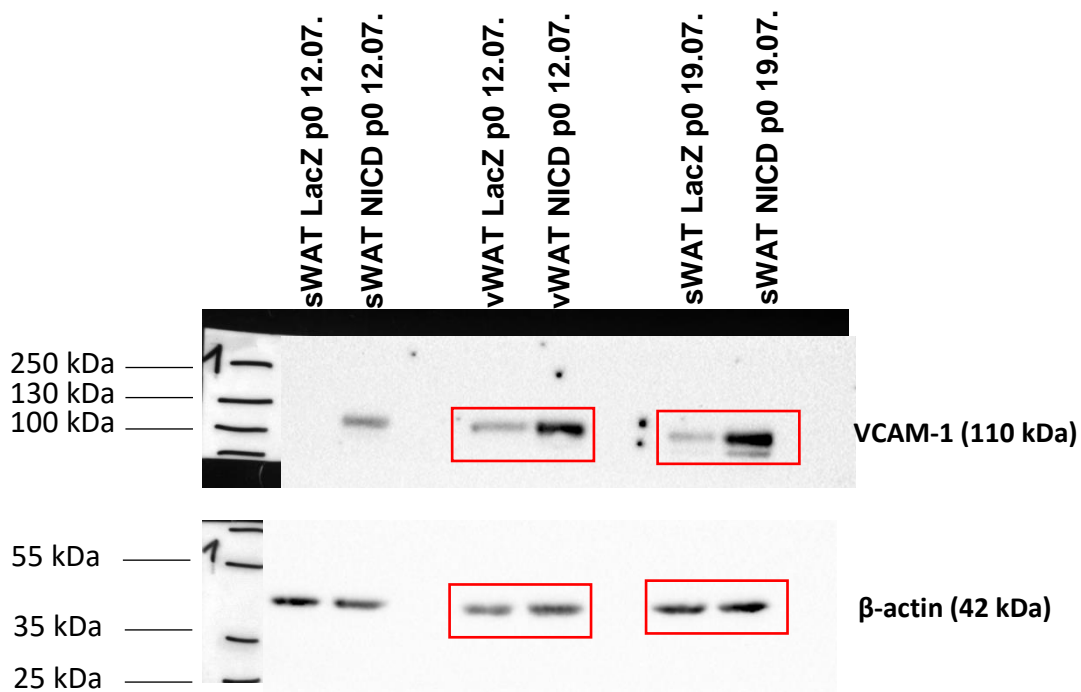

Extended Data Fig. 5o

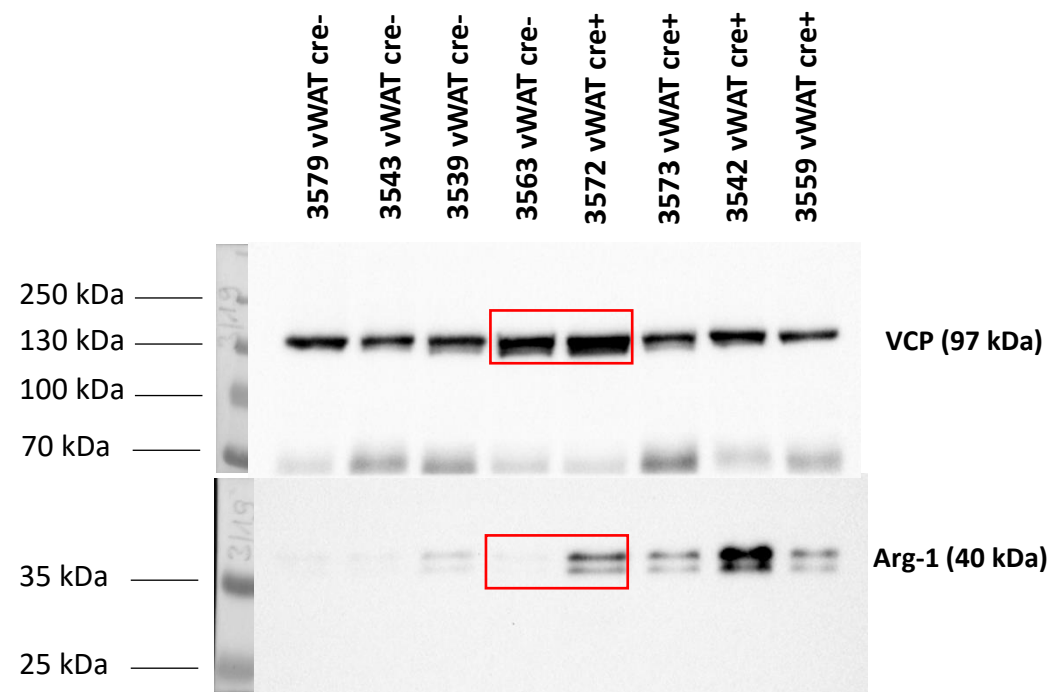

Supplement: Supplementary file 20 — Unprocessed western blots. [file 43018_2023_622_MOESM20_ESM.pdf]
